# Broadening the repertoire of melanoma-associated T-cell epitopes

**DOI:** 10.1007/s00262-015-1664-x

**Published:** 2015-02-18

**Authors:** Thomas Mørch Frøsig, Rikke Lyngaa, Özcan Met, Stine Kiær Larsen, Marco Donia, Inge Marie Svane, Per thor Straten, Sine Reker Hadrup

**Affiliations:** 1 Center for Cancer Immune Therapy, Department of Hematology, University Hospital Herlev, Herlev, Denmark; 2 Department of Drug Design and Pharmacology, Faculty of Health and Medical Sciences, University of Copenhagen, Copenhagen, Denmark; 3 Department of Oncology, University Hospital Herlev, Herlev, Denmark; 4 Present Address: Section for Immunology and Vaccinology, National Veterinary Institute, Technical University of Denmark, Bülowsvej 27, 1870 Frederiksberg C, Copenhagen, Denmark

**Keywords:** T-cell epitope mapping, HLA-A2 negative, Melanoma, MHC multimer, Combinatorial encoding

## Abstract

Immune therapy has provided a significant breakthrough in the treatment of metastatic melanoma. Despite the remarkable clinical efficacy and established involvement of effector CD8 T cells, the knowledge of the exact peptide-MHC complexes recognized by T cells on the tumor cell surface is limited. Many melanoma-associated T-cell epitopes have been described, but this knowledge remains largely restricted to HLA-A2, and we lack understanding of the T-cell recognition in the context of other HLA molecules. We selected six melanoma-associated antigens (MAGE-A3, NY-ESO-1, gp100, Mart1, tyrosinase and TRP-2) that are frequently recognized in patients with the aim of identifying novel T-cell epitopes restricted to HLA-A1, -A3, -A11 and -B7. Using in silico prediction and in vitro confirmation, we identified 127 MHC ligands and analyzed the T-cell responses against these ligands via the MHC multimer-based enrichment of peripheral blood from 39 melanoma patients and 10 healthy donors. To dissect the T-cell reactivity against this large peptide library, we used combinatorial-encoded MHC multimers and observed the T-cell responses against 17 different peptide-MHC complexes in the patient group and four in the healthy donor group. We confirmed the processing and presentation of HLA-A3-restricted T-cell epitopes from tyrosinase (TQYESGSMDK) and gp100 (LIYRRRLMK) and an HLA-A11-restricted T-cell epitope from gp100 (AVGATKVPR) via the cytolytic T-cell recognition of melanoma cell lines and/or K562 cells expressing the appropriate antigen and HLA molecule. We further found T-cell reactivity against two of the identified sequences among tumor-infiltrating lymphocytes from melanoma patients, suggesting a potential clinical relevance of these sequences.

**Electronic supplementary material:**

The online version of this article (doi:10.1007/s00262-015-1664-x) contains supplementary material, which is available to authorized users.

## Introduction

Immune therapy of metastatic melanoma has provided promising clinical results with both long-lasting objective responses and the prolongation of overall survival. The major breakthroughs are based on blocking inhibitory immune-modulating pathways by inhibiting CTLA4 (cytotoxic T-Lymphocyte antigen-4), PD1 (programmed death 1) and PD-L1 (programmed death ligand 1) [1–5]. Furthermore, adoptive cell therapy (ACT) has shown remarkable clinical results, and the implementation of this treatment strategy has spread to several institutions world-wide [6–10]. Although the clinical efficacy of these trials is noteworthy, the characterization of the molecular interactions responsible for tumor cell recognition has been limited. We previously analyzed tumor-infiltrating lymphocyte (TIL) cultures used for ACT for the recognition of all known melanoma-associated T-cell epitopes restricted to human leukocyte antigen (HLA)-A1, -A2, -A3, -A11 and -B7 [11]. We generated a database of 323 described melanoma-associated T-cell epitopes and found that 45 % of these epitopes are restricted to HLA-A2. The remaining epitopes are distributed on 36 different HLA molecules, and the only other well-represented HLA molecule is HLA-A24. Thus, only a few T-cell epitopes have been identified for each of the remaining HLA molecules, which limit our understanding of T-cell recognition in melanoma on a broad population basis.

A number of melanoma-associated antigens are frequently recognized in the context of HLA-A2, including gp100 (glycoprotein 100), Mart1 (melanoma antigen recognized by T-cells 1), NY-ESO-1 (New York Esophagus antigen 1), tyrosinase, TRP-2 (tyrosinase-related protein 2) and some MAGE (melanoma-associated antigen) proteins [11, 12]. The MAGE proteins show considerable sequence similarity, and most T-cell epitopes have been described from MAGE-A3 [11]. Thus, we selected this protein together with the above-mentioned proteins for this T-cell epitope mapping study. Gp100, tyrosinase and TRP-2 are involved in melanin synthesis [13–15], while Mart1 has been found necessary for gp100 function [16], and these proteins are expressed in most melanocytes and melanomas [17–19]. Furthermore, MAGE-A3 and NY-ESO-1 are widely expressed in advanced melanomas [20, 21] and selectively expressed in immune-privileged sites and cancers. To identify novel T-cell epitopes within the amino acid sequence of these antigens, we used a high-throughput T-cell epitope mapping platform [22]. This platform incorporates the production of peptide-MHC (major histocompatibility complex) reagents via the UV light-induced cleavage of conditional ligands [23–25], and the detection of T-cell responses via combinatorial-encoded fluorochrome-coupled MHC multimers [26, 27]. Tumor-associated antigen-specific T cells are rare, and we therefore included an MHC multimer-based T-cell enrichment step prior to detection. The parallel enrichment of up to 400 different specificities is feasible [28], and thus, the method is well suited for the detection of rare T-cell responses in epitope mapping strategies [29].

We successfully identified 17 different CD8 T-cell responses within the peripheral blood from melanoma patients, T cells recognizing four of these were additionally found in the blood from healthy donors. In addition, we screened TIL cultures from 30 melanoma patients with MHC multimers and detected three specific T-cell populations recognizing two of the 17 peptide-MHC complexes. Furthermore, we confirmed the processing and presentation on the surface of target mRNA-electroporated, HLA-transduced K562 cells for two peptides restricted to HLA-A3 and one to HLA-A11. For one T-cell specificity, moreover, we demonstrated the specific killing of a melanoma cell line that could be inhibited by adding cold target cells. The identification of these new T-cell epitopes will provide better tools for immunological monitoring and enable the antigen-specific targeting in HLA-A2-negative individuals.

## Material and methods

### Patient and healthy donor samples

Peripheral blood mononuclear cells (PBMCs) were collected from melanoma patients and healthy donors. The blood samples were drawn a minimum of 4 weeks after the termination of anticancer therapy. The PBMCs were isolated using Lymphoprep (Stemcell Technologies, Grenoble, France) separation, HLA typed (Department of Clinical Immunology, University Hospital, Copenhagen, Denmark) and frozen in fetal calf serum (FCS, from Gibco, Life Technologies, Naerum, Denmark) with 10 % dimethylsulfoxide (Sigma-Aldrich, Broendby, Denmark). All materials used were collected following approval by the Ethics Committee for The Capital Region of Denmark and conducted in accordance with the provisions of the Declaration of Helsinki.

### MHC ligand prediction

NetMHC 3.0 Server [30–32] and NetMCHpan 2.1 Server [33, 34] predicted 9- and 10-mer ligands from the amino acid sequence of the proteins MAGE-A3 (Accession number NM_005362.3), NY-ESO1 (NM_001327.2), gp100 (NM_006928.4), tyrosinase (NM_000372.4) and TRP-2 (NM_001922.3) for binding to HLA-A1, -A3, -A11 and -B7. Peptides with a predicted IC_50_ < 1,000 nM in both servers were purchased and evaluated further.

### UV light-mediated peptide exchange and MHC ELISA

Heavy and light chains were produced in *Escherichia coli* and refolded with a conditional ligand, which was substituted with a peptide of interest upon exposure to UV light (1 h, 366 nm) [23, 24]. To evaluate the affinity of the predicted ligands, the rescue of MHC monomers after UV-induced peptide exchange was analyzed in a sandwich ELISA as previously described [23]. Virus-derived control ligands, HLA-A1/cytomegalovirus (CMV) pp65_YSE_, HLA-A3/influenza NP_ILR_, HLA-A11/Epstein-Barr Virus EBNA4_AVF_ and HLA-B7/CMV pp65_RPH_ and a sample not exposed to UV light were used as positive controls; a sample without the rescue peptide was used as a negative control. Positive control peptides were tested in quadruplicate, and negative controls and test peptides were examined in duplicate. All measurements were repeated, and the absorbance values for test peptides were normalized to control ligands. The selection thresholds are provided in the Supplementary Figure 1. Peptides were purchased from Pepscan Ltd., NL.

### MHC multimer-based enrichment and combinatorial encoding of MHC multimers

Peptide-MHC monomers were multimerized with phycoerythrin (PE)-streptavidin (Biolegend, Nordic Biosite, Copenhagen, Denmark) for MHC multimer-based T-cell enrichment. Briefly, the cells were thawed in 11 ml of RPMI, 10 % FCS (R10) (both from Gibco, Life Technologies, Naerum, Denmark), 25 U/ml DNase (Invitrogen, Life Technologies, Naerum, Denmark) and 2.27 mM MgCl_2_ (Apoteket, Herlev University Hospital, Herlev, Denmark). 1 ml of the cell suspension was irradiated at 25 Gy, washed twice and used as feeder cells. The remaining cells were resuspended in 100 μl R10, pooled PE-coupled MHC multimers were added (0.1 μg/specificity based on MHC complex alone) and the mixture was incubated at 4 °C for 1 h. After careful washing, 20 μl of α-PE beads (Dynabeads Magnetic Beads, Life Technologies, Naerum, Denmark) were added and the cells were left at 4 °C for 15 min, washed, resuspended in 500 μl R10 and applied to MS separation columns (Miltenyi Biotec, Bergisch Gladbach, Germany) according to the manufacturer’s instructions. The trapped cells were flushed out, counted and cultured at 37 °C and 5 % CO_2_, in a 96-well plate (Corning Costar, BD Biosciences, Albertslund, Denmark); each well contained 5,000 positive fraction cells; 50,000 irradiated feeder cells; 11,000 CD3/CD28 activation beads (Dynabeads^®^ Human T-Activator CD3/CD28 for T-cell Expansion and Activation, Life Technologies, Naerum, Denmark); IL-2 (100 IU/ml, Proleukin, Novartis Healthcare, Copenhagen, Denmark) and IL-15 (23.8 IU/ml, Peprotech Nordic, Stockholm, Sweden) [28, 35]. The medium was refreshed every 1–3 days.

After 2–3 weeks of culturing, we tested the cultures for T-cell populations recognizing the peptide-MHC complexes used for enrichment via staining with combinatorial-encoded MHC multimers, as described [26, 27]. In short, MHC monomers were multimerized with two different streptavidin conjugates for each peptide specificity, enabling the simultaneous testing of 36 different specificities in a single sample by combining nine colors into dual-color codes for MHC multimers, in order to measure specific T-cell populations with flow cytometry. The cells were additionally stained with LIVE/DEAD^®^ Fixable Near-IR Dead Cell Stain Kit for 633 or 635 nm excitation (Invitrogen, Life Technologies, Naerum, Denmark), CD8-Alexa Flour 700 (BD Pharmingen, Albertslund, Denmark) or CD8-peridinin chlorophyll (PerCP) (Invitrogen, Life Technologies, Naerum, Denmark) and fluorescein isothiocyanate (FITC) coupled antibodies to CD3 or to a panel of CD4, CD14, CD16, CD19 (all from BD Pharmingen, Albertslund, Denmark) and CD40 (AbD Serotec, Puchheim, Germany). We used the following streptavidin-conjugated fluorochromes to detect the MHC multimer-specific T cells: PE, allophycocyanin (APC), phycoerythrin–cyanin (PE-Cy)7, phycoerythrin-cyanin-based fluorescent dye (PE-CF) 594, Brilliant Violet (BV)421, BV510, BV605, BV650 (all from BioLegend, Nordic Biosite, Copenhagen, Denmark), Quantum dot (Qdot) 585, Qdot 605, Qdot 625, Qdot 655 and Qdot 705 (all from Invitrogen, Life Technologies, Naerum, Denmark). The data were acquired on an LSR II flow cytometer, and the gating strategy is provided in Supplementary Figure 2. Virus-derived epitopes were included as positive controls for the specificity testing but not for the enrichment procedure. All enrichments were repeated (except for one HLA-A1 and one HLA-B7 patient, due to lack of patient material), and all T-cell responses were confirmed in another two-color combination of MHC multimers.

### Sorting of specific T-cell populations

Specific T-cell populations were sorted by fluorescence-activated cell sorting to obtain T-cell cultures with a high frequency of specific T cells. The cells were stained with two-color-coded MHC multimers generated as mentioned above, CD3-FITC (BD Pharmingen, Albertslund, Denmark) and CD8-PerCP (Invitrogen, Life Technologies, Naerum, Denmark). The cells were sorted on an Aria II cell-sorter at 500 cells/well or 3 cells/well into a 96-well plate (Corning Costar, BD Biosciences, Albertslund, Denmark) containing 100,000 irradiated PBMCs (a 1:1:1 mix from three donors), incubated over night with 2 μg/ml phytohemagglutinin (PHA, Gibco, Life Technologies, Naerum, Denmark) in 100 μl of X-vivo 15 (Lonza, Vallensbaek, Denmark), 5 % human serum (Sigma-Aldrich, Broendby, Denmark), IL-2 (500 IU/ml, Proleukin, Novartis Healthcare, Copenhagen, Denmark)), IL-15 (23.8 IU/ml, Peprotech Nordic, Stockholm, Sweden), IL-21 (30 ng/ml, Peprotech Nordic, Stockholm, Sweden) and an antibody to CD3 (OKT3, 0.03 μg/ml, eBioscience, AH Diagnostics, Aarhus, Denmark) and the cells were incubated at 37 °C in 5 % CO_2_. After 5 days, half of the medium was exchanged with X-Vivo 15 containing 5 % human serum, IL-2 (6,000 IU/ml), IL-15 (23.8 IU/ml) and IL-21 (30 ng/ml).

### Production of in vitro transcribed mRNA

cDNA encoding gp100, tyrosinase and TRP-2 was synthesized and cloned into pSP73-SphA64 (kindly provided by Professor E Gilboa, Duke University Medical Center, Durham, NC) using 5`XhoI/3`PacI restriction (Geneart/Life Technologies, Regensburg, Germany). The plasmids were propagated in *E. coli* competent cells (Invitrogen, Paisley, United Kingdom) and purified as described previously [36]. Prior to serving as a DNA template for in vitro transcription, all plasmids were linearized with SpeI restriction enzyme and purified using the Wizard DNA Clean-Up System (Promega, Oslo, Norway). The in vitro transcription was performed as previously described [36].

### Electroporation of K562 cells

The HLA-transduced K562 cells (kindly provided by Mirjam M.H. Heemskerk, Leiden University Medical Centre, the Netherlands) were transfected as previously described [29] with minor modifications. Briefly, HLA-transduced K562 cells were washed twice, resuspended in phosphate-buffered saline (Invitrogen, Life Technologies, Naerum, Denmark) and adjusted to a final cell density of 4 × 10^7^ cells/ml. The cell suspensions (400 μl) were pre-incubated in a 2-mm gap electroporation cuvette for 5 min on ice. mRNA encoding melanoma-associated antigens, gp100: 5.7 μg; tyrosinase: 6.7 μg; TRP-2: 8.9 μg, was used to transfect HLA-transduced K562 cells using a BTX 830 square-wave electroporator (Harvard Apparatus, Holliston MA, USA). The electroporation settings were adjusted to 6 pulses at 560 V for 99 μs. After electroporation, the K562 cells were transferred to preheated R10 medium and rested for 1 h, 37 °C in 5 % CO_2_. The following cells were obtained: K562/HLA-A3/gp100, K562/HLA-A3/tyrosinase, K562/HLA-A11/gp100 and K562/HLA-B7/TRP-2. Reverse Transcriptase PCR confirmed the presence of mRNA after 4, 8 and 24 h of resting at 37 °C.

### VITAL-Far Red cytotoxicity assay

This ‘in vivo/in vitro technique for assessing cell lysis’ (VITAL)-Far Red assay has been described elsewhere [37]. Briefly, HLA-transduced K562 cells electroporated with relevant mRNA were marked with 10 μM carboxyfluorescein succinimidyl ester (CFSE, Invitrogen, Life Technologies, Naerum, Denmark) and incubated for 5 min at 37 °C in 5 % CO_2_; these cells were used as positive target cells. Transduced and not electroporated K562 cells were marked with 5 μM Far Red (Invitrogen, Life Technologies, Naerum, Denmark) and incubated for 5 min at 37 °C in 5 % CO_2_; these cells were used as negative control cells. The target cells and negative control cells were mixed at a ratio of 1:1, 2,000 cells/well in total, in 100 µl R10 in a 96-well plate (Corning Costar, BD Biosciences, Albertslund, Denmark). Specific T cells (effector cells) were added at different ratios to this target cells mixture in 100 μl R10. The cytotoxicity against the positive target cells was measured relative to the cytotoxicity against the negative control cells in the same mixture after 48-h incubation at 37 °C in 5 % CO_2_. Also, a data point without the addition of the effector T cells was included. All measurements were performed in quadruplicate and repeated to confirm the data.

### ^51^Chromium-release cytotoxicity assay

To measure the ^51^Chromium-release, 5 × 10^5^ target cells in 100 µl R10 were labeled with ^51^Cr (100 µCi; Amersham, Arlington Heights, IL) and pulsed with peptide at 37 °C for 1 h when indicated. The washed target cells were incubated with effector cells at various effector/target ratios in 96-well plates at 37 °C for 4 h before 100 µl of medium was aspirated and the ^51^Cr release counted in a gamma counter (Wallac Wizard 1470 Automatic Gamma Counter, Perkin Elmer). The maximum ^51^Cr release was determined in separate wells by adding 100 µl of 10 % Triton X-100, and the spontaneous release was determined by adding 100 µl of R10 to target cells only. The specific lysis was calculated using the following formula: [(experimental release—spontaneous release)/(maximum release—spontaneous release)] × 100. The target cells were melanoma cell lines FM3 (HLA-A3^+^gp100^+^) and FM28 (HLA-A3^−^gp100^+^).

### Generation of TILs

A standard two-step protocol was applied for the generation of clinical grade TIL cultures from tumor fragments, as previously described [38].

## Results

### Mapping of HLA-restricted peptide ligands from melanoma-associated proteins

**Fig. 1:**
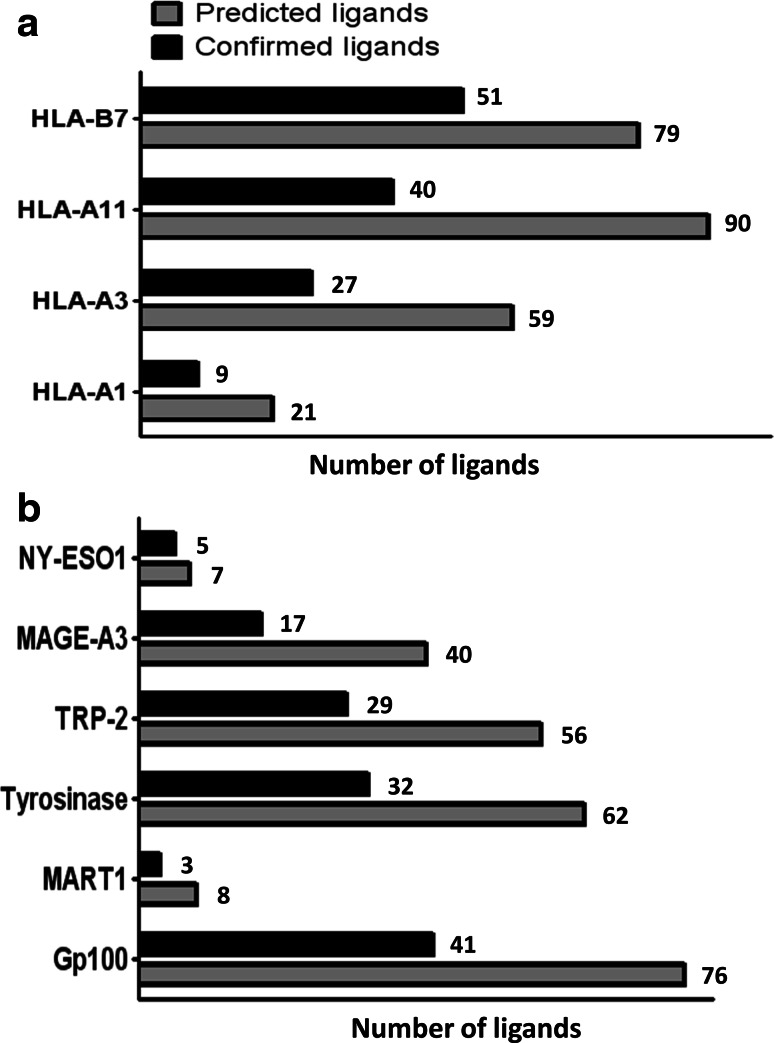
MHC ELISA affinity testing of in silico predicted ligands. Peptides were selected according to the measured absorbance related to the rescue of the specific peptide-MHC complex after UV light-mediated peptide exchange. The absorbance level was calculated relative to a positive control virus-derived peptide and peptides were selected if this normalization value was higher than a given threshold in one of two independent experiments. **a** Shows the number of predicted and confirmed ligands distributed according to HLA restriction, and **b** shows the number of predicted and confirmed ligands distributed according to target the proteins

We predicted 9- and 10-mer peptide ligands for binding to HLA-A1, -A3, -A11 and -B7 from MAGE-A3, NY-ESO-1, gp100, Mart1, tyrosinase and TRP-2 by combining the output from the prediction servers NetMHC [30–32] and NetMHCpan [33, 34]; the threshold for selection was IC_50_ < 1,000 nM for both algorithms. This approach resulted in 249 potential ligands, shown in Supplementary Table 1. These peptides were evaluated experimentally for their binding affinity to the relevant HLA molecules, measured by MHC ELISA as the peptide-dependent MHC rescue after the UV light-mediated exchange of a conditional ligand [23–25]. The peptides were selected based on their ability to stabilize HLA molecules in either of two independent experiments, and the number of predicted and confirmed ligands for each allele is shown in Fig. 1a; data on the binding affinity of each potential ligand is depicted in Supplementary Figure 1 with gray illustrating the selected ligands. From the 249 predicted HLA ligands, we selected 127 confirmed ligands for further assessment in T-cell recognition analyses. The protein distribution of these ligands is shown in Fig. 1b; differences between the numbers of predicted peptides reflect differences in the protein size.

### Mapping of melanoma-associated T-cell responses

**Fig. 2:**
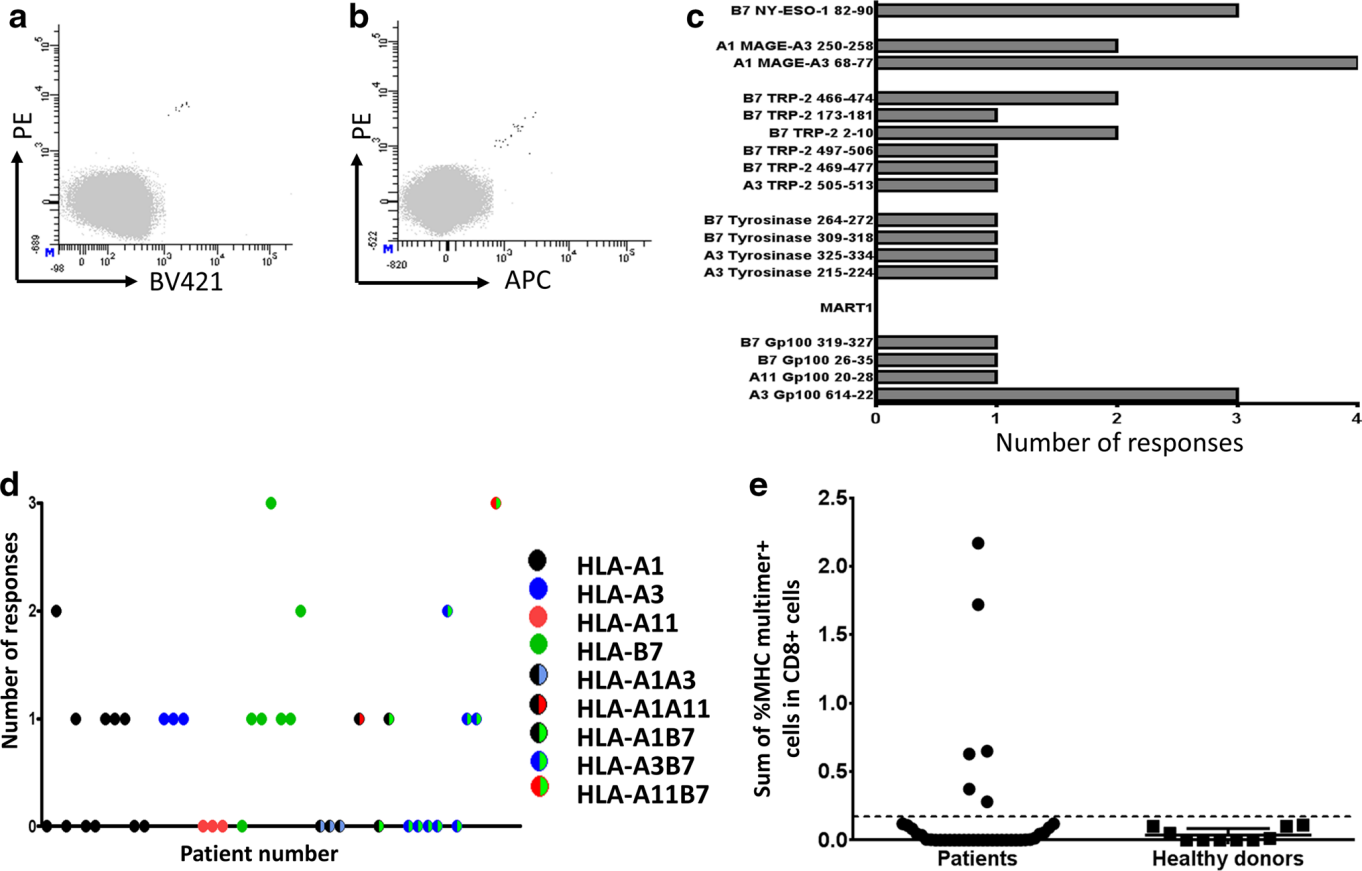
Detection of immune responses after MHC multimer-based enrichment. **a**, **b** Example of a confirmed response found with combinatorial encoding of MHC multimers detecting HLA-A3 Gp100_614-22_ reactive T cells, after a PE-MHC multimer-based enrichment of PBMCs. **a** 0.004 % of 270,000 CD8 cells detected in the PE/BV421 MHC multimer combination. **b** 0.004 % of 510,000 CD8 cells detected in the PE/APC MHC multimer combination. Only CD8 cells negative for all MHC multimer (*gray*) or specific for exactly two MHC multimers (*black*) are shown, the gating strategy is provided in Supplementary Figure 2. **c** The total number of T-cell responses detected per peptide sequence, grouped according to their protein origin. **d** The total number of responses detected per patient, when plotted according to patients’ HLA type. Each *dot* represents one patient. **e** The sum of melanoma-associated MHC multimer-specific T cells detected for each patient/healthy donor. The *dotted line* represents the mean frequency of responses found in the heathy donor cohort+ 3*standard deviation. Responses with higher frequency in the patient cohort are: HLA-B7/gp100_319-327_, HLA-B7/gp100_26-35_ and HLA-B7/NY-ESO_82-90_; HLA-A1/MAGE-A3_68-77_ (2 responses); and HLA-A3/gp100_614-22_

We used the library of 127 confirmed HLA ligands to screen the peripheral blood cells from 39 melanoma patients to detect specific T-cell populations. We included an MHC multimer-based enrichment step prior to the detection with the aim of enhancing the specific T-cell frequency, as spontaneous tumor-associated T-cell responses are often scarce and predominantly expected to be below the threshold of the detection method [29]. We previously established the detection limit for the detection of specific T-cell responses by combinatorial-encoded MHC multimers to be 0.002 % of the CD8 cells with at least 10 MHC multimer positive events [27]. We generated a library of PE-labeled peptide-MHC multimers and used relevant complexes based on the patients’ HLA type to positively select HLA multimer-binding T cells over a magnetic column via capture with anti-PE-coated magnetic beads. The cells were cultured for 2–3 weeks prior to the testing of specific peptide-MHC recognition by using combinatorial-encoded MHC multimers [27]. Each culture was tested for the presence of T-cell populations specific for the peptides used for T-cell enrichment as well as two virus-derived T-cell epitopes for each HLA molecule. We confirmed all detected responses with another MHC multimer staining using a different color-code, as exemplified for one response in Fig. 2a, b.

A total of 27 T-cell populations recognizing 17 different peptide sequences in complex with the appropriate HLA molecules from five of the six proteins were detected in the PBMCs from 39 patients. An overview of all T-cell responses is provided in Table 1 and dot plots of the different responses are provided in Supplementary Figure 3. The T-cell responses against any of the included sequences were detected in 20/39 patients (51 %), irrespectively of the patients’ HLA type. Differences in the protein size and number of HLA ligands from each protein hinder the direct comparison of the response rates, but overall we most frequently detected responses against the TRP-2-derived peptides (8/39 patients). In addition, the majority of recognized peptides stem from three of the differentiation antigens. No Mart1-specific responses were detected. Figure 2c shows the number of T-cell responses detected for each peptide/protein. Figure 2d illustrates the number of responses in each patient according to HLA type, and it is evident that all HLA molecules are involved in the recognition of melanoma-associated peptides, but most responses were detected in conjunction with HLA-B7 with 14 responses in 17 tested HLA-B7-positive patients.

**Table 1:** Information about 27 (17 different) T-cell responses detected in 39 melanoma patients

Protein	Sequence	Amino acid position	Tissue-type restriction	Detection rate	Validation of epitope processing in electroporated, HLA-transduced K562 cells	Detection of T-cell responses in TIL cultures
MAGE-A3	ASSLPTTMNY	68–77	HLA-A1	4/17^a^	ND	0/18
	FVQENYLEY	250–258	HLA-A1	2/17	ND	0/18
NY-ESO1	GPESRLLEF	82–90	HLA-B7	3/18	ND	0
TRP-2	LMETHLSSK	505–513	HLA-A3	1/14	ND	0/16
	SPLWWGFLL	2–10	HLA-B7	2/18	No	0
	QPQFANCSV	173–181	HLA-B7	1/18	ND	0
	TPGWPTTLL	466–474	HLA-B7	2/18	ND	2
	WPTTLLVVM	469–477	HLA-B7	1/18	ND	0
	RLRKGYTPLM	497–506	HLA-B7	1/18	No	1
Tyrosinase	LLRWEQEIQK	215–224	HLA-A3	1/14	ND	0/16
	SPASFFSSW	264–272	HLA-B7	1/18^a^	ND	0
	TPRLPSSADV	309–318	HLA-B7	1/18	ND	0
	TQYESGSMDK	325–334	HLA-A3	1/14	Yes	0/16
Gp100	AVGATKVPR	20–28	HLA-A11	1/5	Yes	0/4
	VPRNQDWLGV	26–35	HLA-B7	1/18^a^	ND	0
	APNTTAGQV	319–327	HLA-B7	1/18	ND	0
	LIYRRRLMK	614–622	HLA-A3	3/14^a^	Yes	0/16
Total number of responses				27		3

*ND* not determined, *TIL* tumor-infiltrating lymphocyte. Most TIL cultures were not HLA typed for HLA-B7

^a^T-cell specificities also detected in the healthy donor cohort

We further assessed if these melanoma-associated T-cell responses could be detected directly ex vivo in patient PBMCs. We investigated nine patients for the presence of 13 T-cell specificities, which were detected after the MHC multimer-based T-cell enrichment of their PBMC samples, including a number of more frequent responses, ranging from 0.1 to 2.2 % of the CD8 T cells. However, no T-cell responses against melanoma-associated proteins were found in this screening, although we did observe T-cell responses against virus-derived peptides for 6/9 patients (data not shown). This indicates that PBMCs hold very low numbers of T cells recognizing these melanoma-associated T-cell epitopes and the T-cell enrichment strategy is therefore required for detection, as illustrated in Supplementary Figure 4a and b.

To investigate whether these T-cell responses would be selectively found in melanoma patients, we additionally screened blood from 10 healthy donors via the MHC multimer-based enrichment for T-cell reactivity against any of the 17 peptide-MHC complexes found to be recognized in patient blood. We found a total of six responses against 4 peptide-MHC complexes present in 5/10 healthy donors (Table 1, indicated by superscript letter a). There was no overall difference in the frequency of response between the two groups (20/39 melanoma patients and 5/10 healthy donors). However, in the patient group, a number of individuals presented with a higher frequency of multimer-specific T cells than in the healthy donor group (Fig. 2e).

### Recognition of endogenously processed tumor-associated antigens

**Fig. 3:**
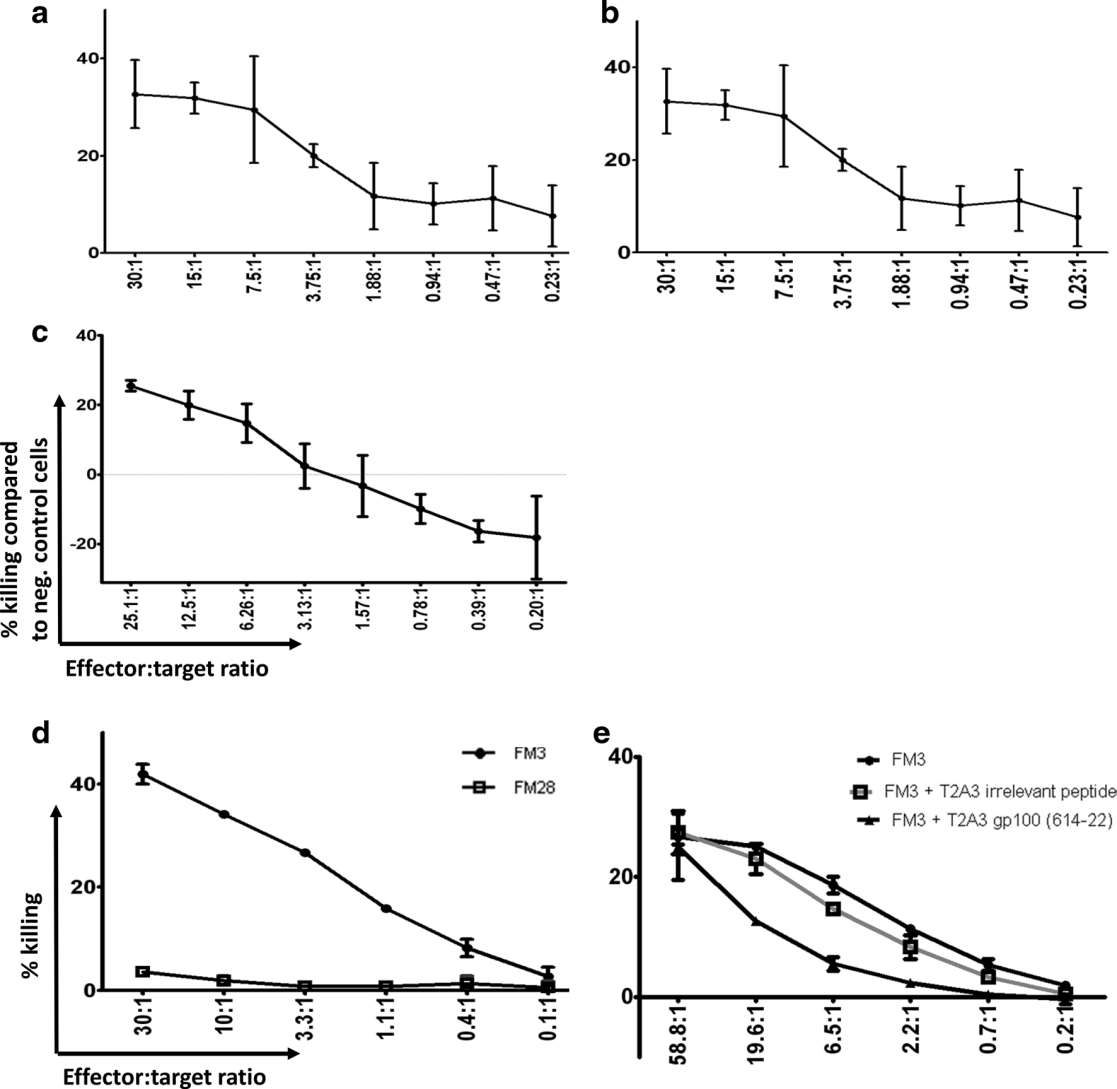
Recognition of processed antigen in K562 cells or melanoma cell lines. **a** Lysis of K562/HLA-A3/gp100 cells compared with K562/HLA-A3 cells when co-cultured with increasing numbers of HLA-A3 gp100_614-622_-specific T cells (60.0 % of CD8 cells) in the given specific effector/target cell ratio, determined as the ratio between MHC multimer positive T cells and K562/HLA-A3/gp100 cells. **b** Lysis of K562/HLA-A3/tyrosinase cells compared with K562/HLA-A3 cells when co-cultured with increasing numbers of HLA-A3/tyrosinase_325-334_-specific T cells (81.7 % of CD8 cells) in the given effector:target cell ratio. **c** Lysis of K562/HLA-A11/gp100 cells compared with K562/HLA-A11 cells when co-cultured with increasing amounts of an HLA-A1/gp100_20-28_-specific T-cell culture (51.8 % of CD8 cells) in the given effector/target cell ratio. **d** Chromium-release cytotoxicity testing of the HLA-A3/gp100_614-622_ culture (60.0 % of CD8 cells) against gp100 positive and HLA-A3-positive (FM3) and HLA-A3-negative (FM28) melanoma cell lines. **e** Cytolytic recognition of the FM3 melanoma cell line and cold target inhibition of gp100_614-22_-specific T cell-mediated killing with HLA-A3-transduced T2 pulsed with either gp100_614-22_ or an irrelevant tyrosinase-derived peptide. Data shown in (**a**–**c)** were measured with the VITAL-Far Red assay after 48 h of co-culturing of effector and target cells at 37 °C, and data shown in (**d**, **e)** were obtained with a standard Cr^51^-release assay after 4 h of co-culturing of effector and target cells at 37 °C. Shown in all plots is the relative lysis of positive target cells. The effector/target ratio is calculated based on MHC multimer-specific effector T cells.* Bars* represent the standard deviation of two independent experiments

We successfully sorted and short-term expanded T-cell populations specific for five different peptide sequences from gp100 (2), tyrosinase (1) and TRP-2 (2). These T-cell cultures were used to confirm the recognition and antigen processing using HLA-transduced and antigen mRNA-transfected K562 cells. We determined the T cell-mediated killing of target cells after co-culture with T cells using a VITAL-Far Red cytotoxicity assay. MHC multimer-specific T cells were added in increasing ratios to the number of antigen-transfected HLA-transduced K562 cells, and specific killing was measured relative to non-transfected HLA-transduced K562 cells. Three specific cultures recognizing HLA-A3/gp100_614-622_, HLA-A3/tyrosinase_325-334_ or HLA-A11/gp100_20-28_ showed antigen-specific and effector:target ratio-dependent killing of target cells (Fig. 3a–c). We were not able to obtain a functional readout for the additional two cultures (Table 1). Additionally, we measured the T cell-mediated killing of melanoma cell lines, FM3 (HLA-A3^+^gp100^+^) and FM28 (HLA-A3^−^gp100^+^) via co-culturing with HLA-A3-restricted gp100_614-622_-specific T cells (Fig. 3d). We observed the selective killing of the HLA-A3-positive melanoma cell line FM3, and this killing was specifically inhibited by cold target inhibition with the addition of T2-A3 cells pulsed with gp100_614-622_, whereas it was not affected by addition of T2-A3 cells pulsed with an irrelevant peptide (Fig. 3e). Furthermore, T2-A3 cells pulsed with gp100_614-622_ or tyrosinase_325-334_ were specifically killed by T cells with matching specificities (Supplementary Figure 5a and b, respectively).

### Screening of immune reactivity in melanoma TILs

**Fig. 4:**
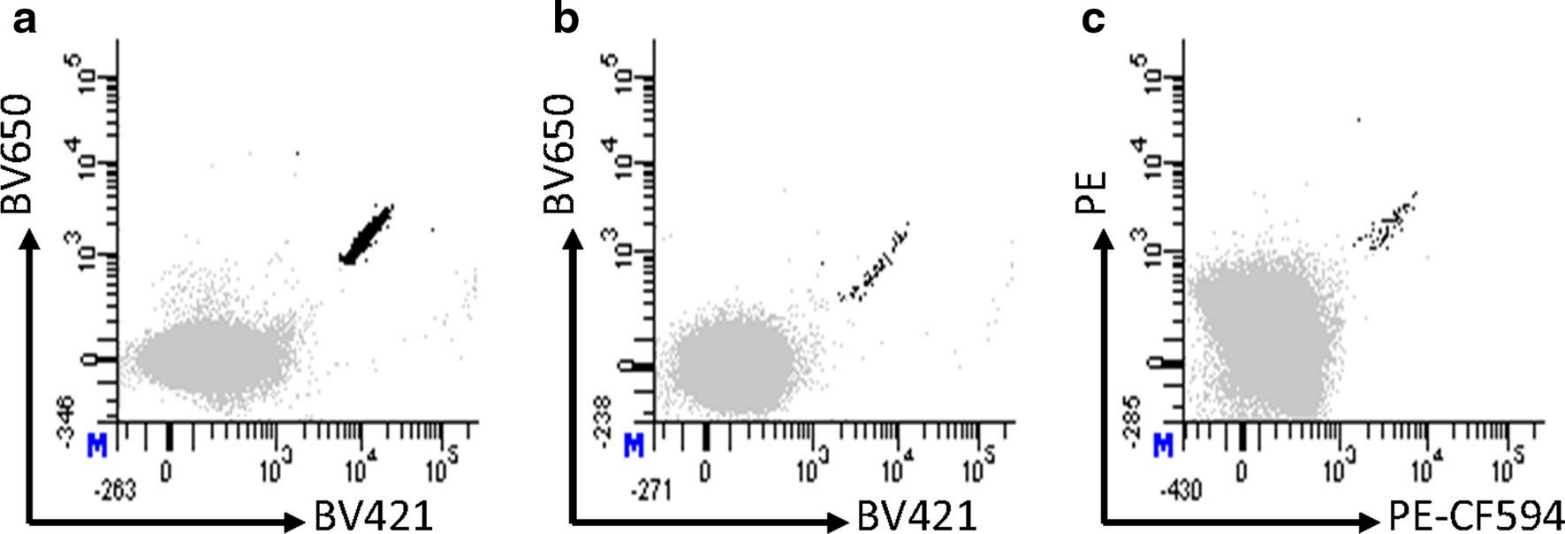
T-cell responses in melanoma TILs detected by dual-color-coded MHC multimers. **a** 0.41 % HLA-B7/TRP-2_466-474_-specific T cells of 609.000 CD8 cells detected with the BV421:BV650 MHC multimer combination. **b** 0.037 % HLA-B7/TRP-2_466-474_-specific T cells of 176.000 CD8 cells detected with the BV421:BV650 MHC multimer combination, **c** 0.017 % HLA-B7/TRP-2_497-506_-specific T cells of 366.000 CD8 cells detected with the PE:PE-CF594 MHC multimer combination. All responses were additionally confirmed using another two-color combination

To further investigate the clinical relevance of the 17 T-cell specificities detected after MHC multimer-based enrichment of PBMCs, we screened TIL cultures generated from 30 melanoma patients (18 HLA-A1^+^, 16 HLA-A3^+^, 4 HLA-A11^+^, 3 HLA-B7^+^ and 27 with unknown HLA-B7 status) for the presence of T-cell populations, recognizing any of these or three virus-derived peptides (HLA-A1/CMV pp50_VTE_, HLA-A3/CMV pp150_VTT_ and HLA-B7/CMV pp65_RPH_). We detected melanoma-associated T-cell responses in three of these patients; two responses against HLA-B7/TRP-2_466-474_ and one against HLA-B7/TRP-2_497-506_, in parallel with a total of 6 virus-specific T-cell responses (Fig. 4; Table 1). Thus, this extended cohort of melanoma-associated T-epitopes may add to our understanding of the tumor recognition element seen in melanoma-infiltrating lymphocytes used for ACT. All the analyzed TIL cultures were generated for clinical use; however, only one of the three patients holding a TRP-2 T-cell response were treated with ACT; this patient experienced a partial response to therapy. None of these patients were included in the initial screening of the PBMC samples.

## Discussion

Previously, T-cell epitope mapping studies in melanoma have focused on HLA-A2 and HLA-A24, and only few T-cell epitopes restricted to other tissue types have been published [11]. The knowledge obtained in this study, of T-cell reactivity against 17 different peptide sequences derived from MAGE-A3 (2), NY-ESO-1 (1), gp100 (4), tyrosinase (4) and TRP-2 (6), will help broadening the measurements of the T-cell repertoire restricted to HLA-A1, -A3, -A11 and -B7, even though we have only formally confirmed a minority of these as T-cell epitopes.

We specifically assessed the processing and presentation for a part of these sequences and showed T-cell recognition on the surface of target mRNA-electroporated HLA-transduced K562 cells for three of the five peptides tested. The processing and presentation of the remaining peptide sequences were not tested due to an insufficient number of specific T cells. However, we do anticipate based on the experience that the majority of these sequences will be presented on the cell surface. To fully confirm the processing and presentation for all peptides in Table 1, further cytotoxicity studies including sequence-specific inhibition are needed for the remaining 11 peptide sequences. HLA-A3/gp100_614-22_-specific T cells further showed cytolytic activity toward a melanoma cell line in an HLA and protein-specific manner. HLA-A3/Tyrosinase_325-334_ and HLA-A11/gp100_20-28_ are newly identified T-cell epitopes, while HLA-A3/gp100_614-22_ was published previously [39]. However, in this previous study, the T-cell recognition of HLA-A3/gp100_614-22_ on the surface of melanoma cells was measured based on the secretion of interferon-γ and our study is the first to show the direct T cell-mediated killing of cells that present this epitope on the surface. The confirmation of this gp100-derived T-cell epitope also served as an internal control for our T-cell epitope mapping study. Additionally, it is one of the most frequently recognized responses in our study; we detected HLA-A3/gp100_614-22_-specific T cells in 3/14 (21 %) tested HLA-A3 positive patients, suggesting its relevance as a broadly recognized melanoma-associated T-cell epitope.

A number of responses were also detected in the healthy donor cohort (Fig. 2e; Table 1), which likely relates to the self-origin of the analyzed peptide sequences, combined with the sensitivity of our detection method. Using the same methodology, we recently showed a complete lack of T-cell recognition of oncogenic proteins from the Merkel cell polyoma virus in a healthy donor cohort, while these were frequently recognized in a Merkel cell carcinoma cohort. Additionally, both cohorts harbored reactivity toward virus capsid proteins [29]. Hence, we believe the T-cell responses found in the current study originate from antigen-experienced cells potentially relevant in establishing anti-tumor reactivity and not from the naïve repertoire. Previously reactivity against melanoma-associated differentiation antigens present in the blood from healthy donors has mainly been described in relation to the HLA-A2/Mart1_26-35_ epitope [40]. Also Tyrosinase-reactive T cells have previously been found in the blood from healthy donors [41], while spontaneous healthy donor CD8-mediated T-cell reactivity against gp100 and MAGE-A3 has to our knowledge never been described before.

We did not detect any responses to peptides from the Mart1 protein, which is striking given the frequent detection of T cells specific for the HLA-A2/Mart1_26-35/27-35_ epitope, e.g., 21/30 melanoma TIL cultures in a previous study showed HLA-A2/Mart1_26-35/27-35_ reactivity [11]. It has been shown that peptides overlapping this region could also be presented to T cells by HLA-B44, HLA-B45 and HLA-B35 molecules [42, 43], whereas the mapping of epitopes from other regions of the protein has failed to a large extent, with the exception of the identification of Mart1_51-61_ as an HLA-Cw7-restricted epitope [44]. The exceptional high frequency of MART-1_26-35_-reactive T cells in both patients and healthy individuals has been explained by a combination of a preferential pairing of the MART-1_26-35_ T-cell receptor [45], an excessive positive selection owing to the cross-reactivity to other self-antigens expressed in cortical thymic epithelial cells [46], and the mis-initiated transcription of *MART*-*1* in medullary thymic epithelial cells resulting in inefficient negative selection [47].

We additionally screened TIL cultures from melanoma patients and found responses in three patients against two peptide sequences derived from TRP-2 and restricted to HLA-B7. The level of reactivity is comparable with our previous finding with mapping of T-cell reactivity in melanoma TILs toward a large panel of melanoma-associated T-cell epitopes [11, 12]. The response rate in our study, when calculated as the number of detected responses compared with the theoretical maximal number of responses ignoring HLA-differences of the patient cohort (3 responses out of 17 peptides × 30 cultures), was 0.6 %, while in the previous study the similar number was 0.8 % [11]. However, this value included responses against the very frequently detected HLA-A2/Mart1_26-35(27L)_ epitope, and as mentioned above, the T-cell recognition of this epitope is special. If these responses are left out the response rate in the previous study also yields a value of 0.6 % [11]. Two of the TIL cultures holding a TRP-2 response in the present study where additionally screened using the larger peptide library (as in [11]), revealing additionally two responses. Thus, the present findings add to the notion that these peptides increase our understanding of antigen recognition in these cultures. Anti-tumor reactivity in expanded TILs is associated with clinical benefit [48]; and selection of the TILs toward cancer-associated antigen-specific cells prior to infusion may improve the clinical efficacy of this strategy. The extended repertoire of the relevant epitopes, as presented here, will broaden the possible implementation of such strategies, independent of patient’s HLA type, and extend our possibility to define the most essential T-cell epitopes.

Antibodies targeting CTLA4 and PD-1/PD-L1 have shown remarkable clinical efficacy in metastatic melanoma [1, 2, 5]. These therapies may depend on an existing endogenous immune response, and although anti-tumor immunity can be stimulated in a subgroup of patients, we do not know to what extent the remaining patients harbor tumor-reactive T cells. To select immunocompetent patients with preexisting immune responses that are more likely to respond clinically, comprehensive monitoring strategies, ideally covering the majority of HLA alleles expressed, are needed. The T-cell responses and epitopes we report here will contribute to a better understanding of anti-melanoma T-cell immunity and possibly enhance the new initiatives toward improved clinical efficacy of the immune therapeutic treatment of metastatic melanoma.

### Electronic supplementary material

Below is the link to the electronic supplementary material.

Supplementary material 1 (PDF 408 kb)

## Abbreviations

ACT: Adoptive cell therapy
APC: Allophycocyanin
BV: Brilliant violet
CMV: Cytomegalovirus
CTLA4: Cytotoxic T-Lymphocyte antigen-4
FCS: Fetal calf serum
FITC: Fluorescein isothiocyanate
Gp100: Glycoprotein 100
HLA: Human leukocyte antigen
VITAL: In vivo/in vitro technique for assessing lysis
MAGE: Melanoma-associated antigen
MHC: Major histocompatibility complex
Mart1: Melanoma antigen recognized by T cells 1
NY-ESO-1: New York Esophagus antigen 1
PBMCs: Peripheral blood mononuclear cells
PD1: Programmed death 1
PD-L1: Programmed death ligand 1
PE: Phycoerythrin
PE-Cy: Phycoerythrin–cyanin
PE-CF: Phycoerythrin–cyanin-based fluorescent dye
PerCp: Peridinin chlorophyll
Qdot: Quantum dot
R10: RPMI supplemented with 10 % FCS
TIL: Tumor-infiltrating lymphocyte
TRP-2: Tyrosinase-related protein 2
