# Supplementary material for: Broadening the repertoire of melanoma-associated T-cell epitopes
Source: Cancer Immunol Immunother. 2015 Feb 18;64(5):609–20. doi: 10.1007/s00262-015-1664-x (PMC4412285; doi:10.1007/s00262-015-1664-x)
Supplement: Supplementary file 1 — Supplementary material 1 (PDF 408 kb) [file 262_2015_1664_MOESM1_ESM.pdf]

Supplementary Material for Frøsig et al,

Cancer Immunology, Immunotherapy

Supplementary Figure 1

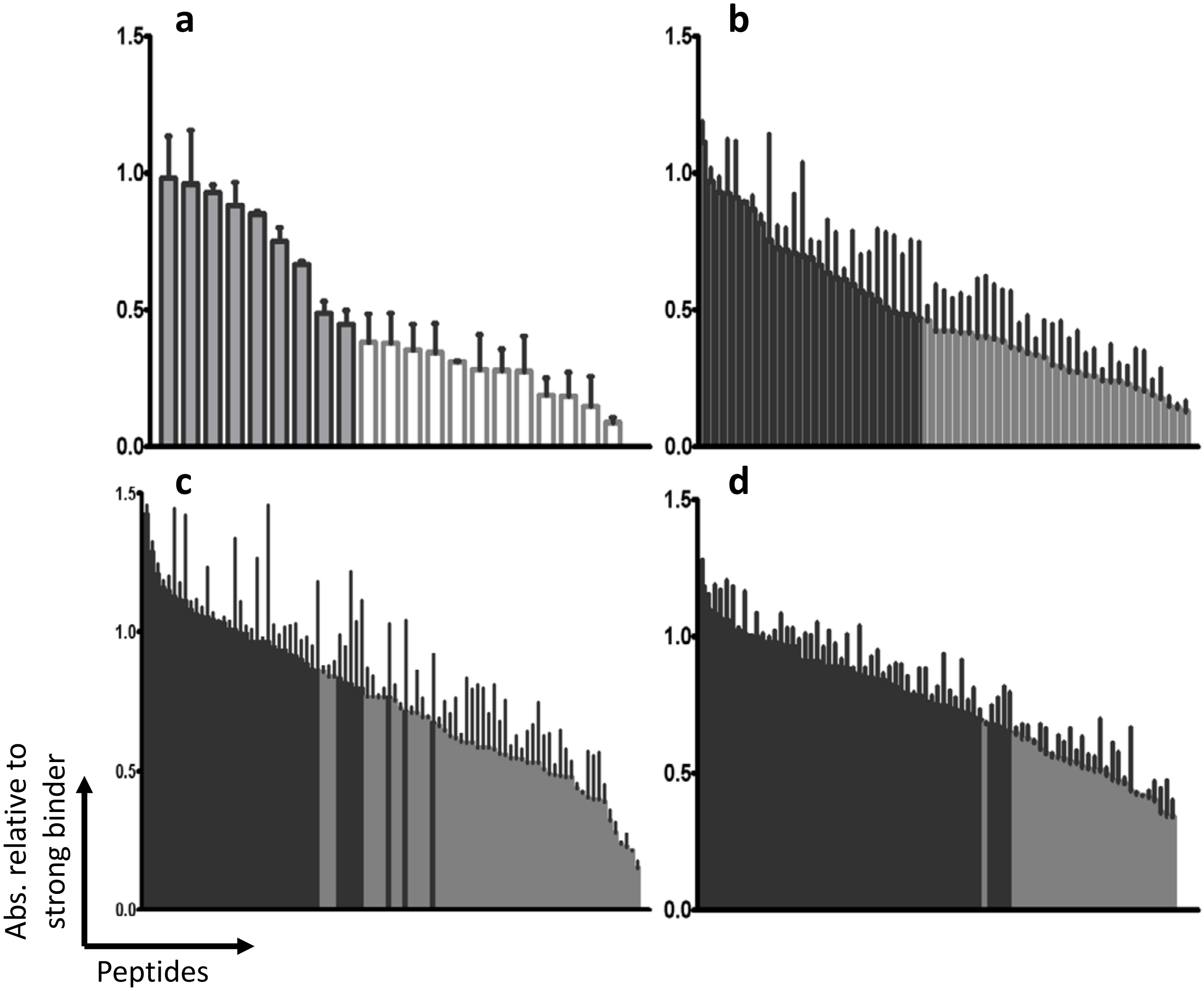

Supplementary Figure 1 Testing via MHC ELISA of the MHC binding affinity of the predicted HLA ligands. The average normalized affinity of each peptide relative to a virus-derived high-affinity ligand is provided; gray bars indicate the peptides selected for further analysis. Peptides were selected if they had a measured affinity above threshold in either of two experiments. Therefore, the group of selected peptides it not exclusively the peptides with highest average normalized affinity. The selection thresholds were 0.49 of the control ligand absorbance for both HLA-A1 peptide affinity measurements; 0.63 and 0.50 of the control ligand absorbance for HLA-A3 peptide affinity measurements, respectively; 0.90 of the control ligand absorbance for both HLA-A11 peptide affinity measurements and 0.70 of the control ligand absorbance for both HLA-B7 peptide affinity measurements. **a)** The 21 predicted HLA-A1 ligands tested and 9 peptides selected, control ligand HLA-A1/CMV pp65$_{YSE}$. **b)** The 59 predicted HLA-A3 ligands tested and 27 peptides selected; control ligand HLA-A3/FLU NP$_{ILR}$. **c)** The 90 predicted HLA-A11 ligands tested and 40 peptides selected; control ligand HLA-A11/EBV EBNA4A$_{AVF}$. **d)** The 79 predicted HLA-B7 ligands tested and 51 peptides selected; control ligand HLA-B7/CMV pp65$_{RPH}$. The bars represent the standard deviation from two experiments.

Supplementary Figure 2

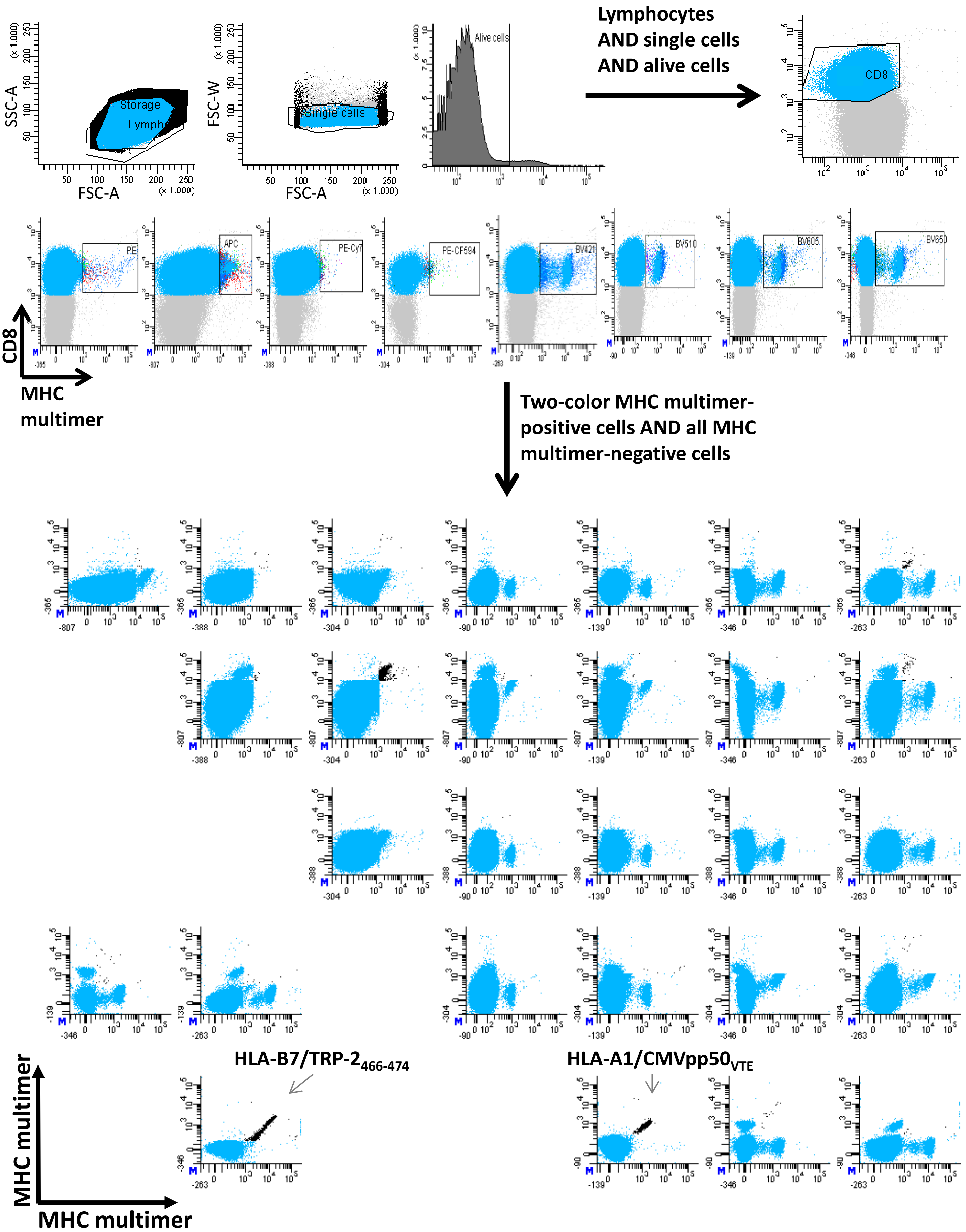

Supplementary Figure 2 The gating strategy used for the identification of dual-colored specific T-cell populations with combinatorial encoded MHC multimers. Shown are dot plots from a representative experiment. From all recorded cells we gated on the lymphocytes, the single cells and the live cells. From the combination of these we gated on the CD8 cells and subsequently the MHC multimer^{+} cells for each of the fluorochromes used for MHC multimer staining. These were combined with the appropriate inverse gates for each of the MHC multimer-fluorochromes, and in the MHC multimer-combination plots are only shown cells gated within two or none MHC multimer gates. Depicted are two specific T-cell populations: HLA-B7/TRP-$2_{466\text{-}474}$ in the BV421:BV650 combination and HLA-A1/CMVpp50_{VTE} in the BV605:BV650 combination. The remaining double-positive cells (black) were not confirmed in a second staining. A: areal of peak, FSC: forward scatter, SSC: side scatter. BV: Brilliant Violet. BV421, BV605 and BV650 are all fluorochrome-coupled MHC multimers.

Supplementary Figure 3

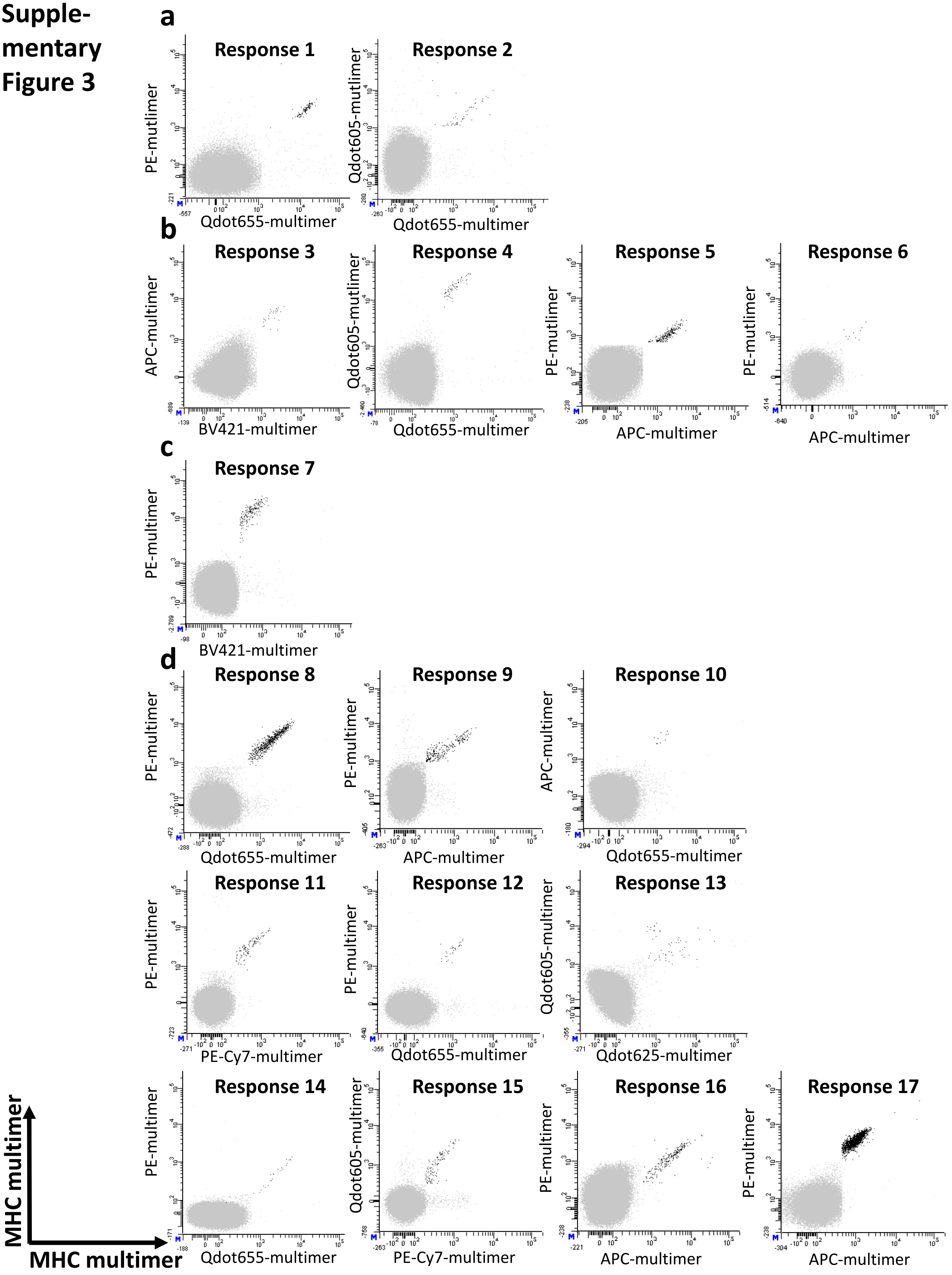

Supplementary Figure 3 Representative dot plots of dual color-coded MHC multimers for each of the 17 different T-cell responses detected in the study, sorted according to HLA-restriction. **a)** 2 HLA-A1 restricted responses, response 1: HLA-A1/MAGE-A3$_{68-77}$, 0.019% of CD8 cells; response 2: HLA-A1/MAGE-A3$_{250-258}$, 0.017% of CD8 cells. **b)** 4 HLA-A3 restricted responses, response 3: HLA-A3/tyrosinase$_{215-224}$, 0.006% of CD8 cells; response 4: HLA-A3/gp100$_{614-622}$, 0.044% of CD8 cells; response 5: HLA-A3/tyrosinase$_{325-334}$, 0.10% of CD8 cells; response 6: HLA-A3/TRP-2$_{505-513}$, 0.003% of CD8 cells. **c)** 1 HLA-A11 restricted response, response 7: HLA-A11/gp100$_{20-28}$, 0.091% of CD8 cells. **d)** 10 HLA-B7 restricted responses, response 8: HLA-B7/gp100$_{319-327}$, 0.28% of CD8 cells, response 9: HLA-B7/TRP-2$_{173-181}$,0.062% of CD8 cells; response 10: HLA-B7/tyrosinase$_{309-318}$, 0.003% of CD8 cells; response 11: HLA-B7/tyrosinase$_{264-272}$,0.036% of CD8 cells; response 12: HLA-B7/TRP-2$_{497-506}$, 0.015% of CD8 cells; response 13: HLA-B7/TRP-2$_{469-677}$, 0.031%; response 14: HLA-B7/TRP-2$_{466-474}$, 0.002% of CD8 cells; response 15: HLA-B7/TRP-2$_{2-10}$, 0.048% of CD8 cells; response 16: HLA-B7/NY-ESO-1$_{82-90}$, 0.47% of CD8 cells; response 17: HLA-B7/gp100$_{26-35}$, 2.17% of CD8 cells. Given on the X- and Y-axes are fluorochome-conjugated MHC multimers for each dot plot. Only CD8 cells negative for all MHC multimers (gray) or positive for exactly two MHC multimers (black) are shown. Qdot: quantum dot, BV: brilliant violet.

Supplementary Figure 4

a

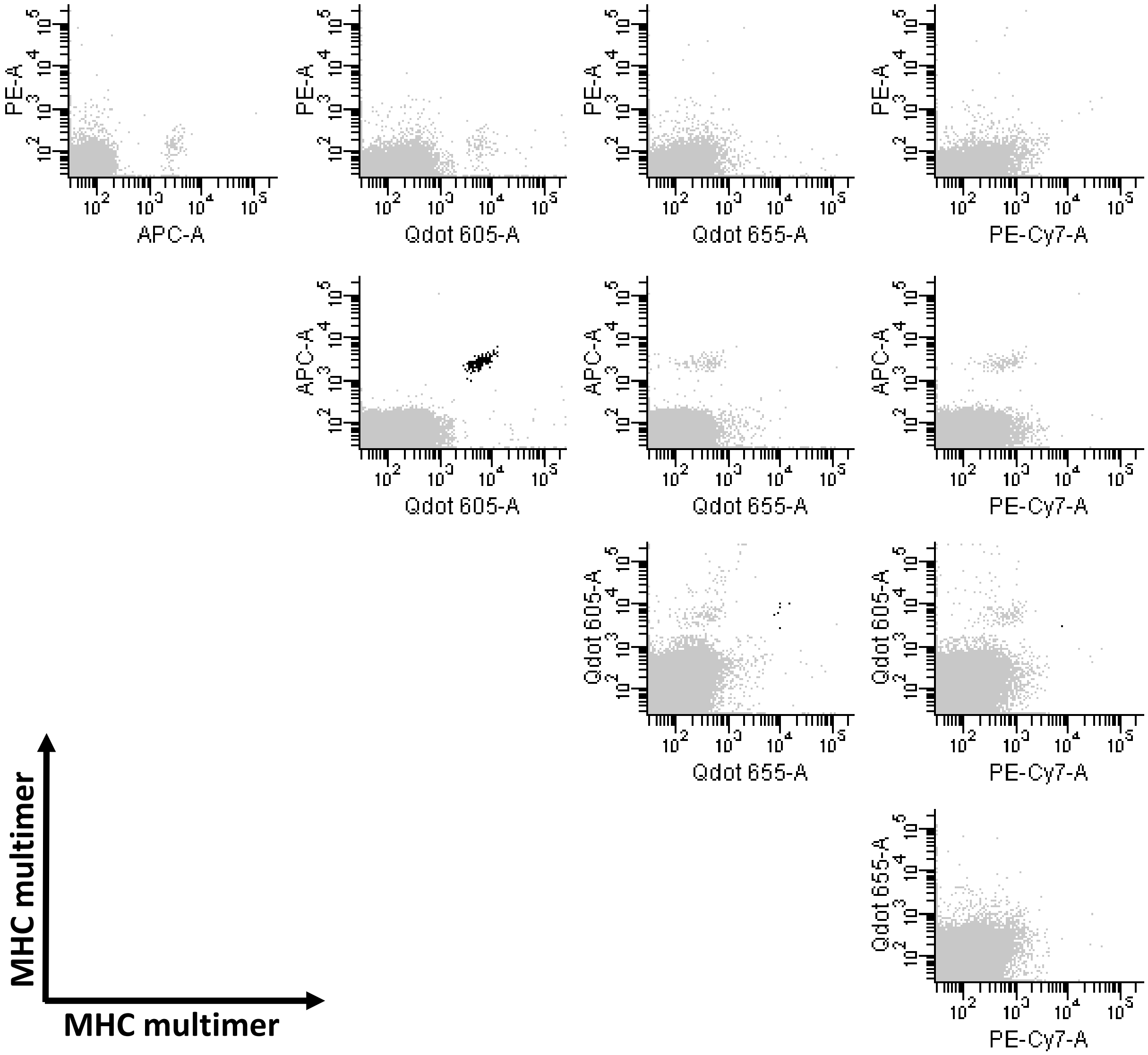

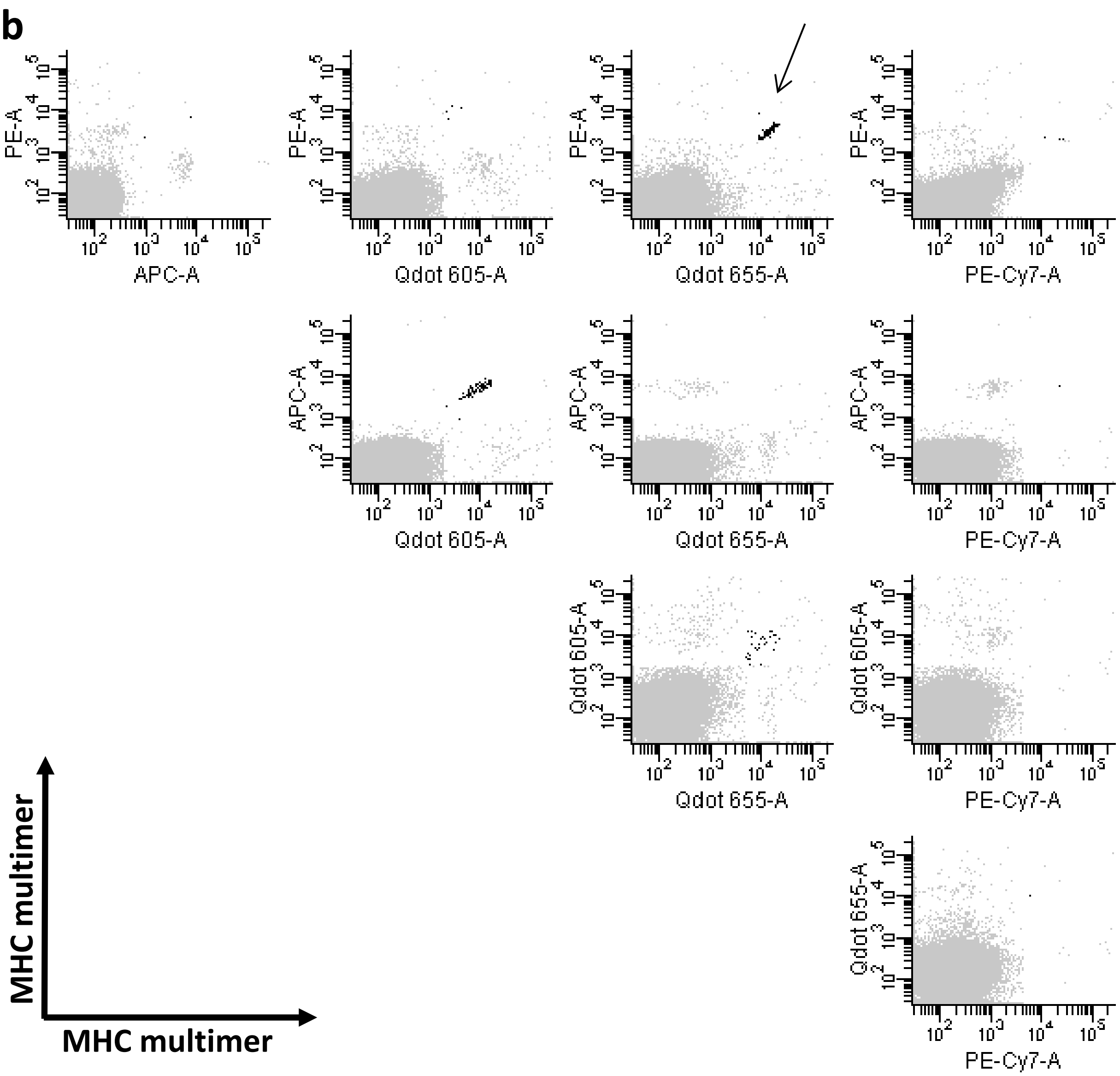

Supplementary Figure 4 The MHC multimer-based enrichment of a T-cell response specific for MAGE-A3$_{68\text{-}77}$, coded in the PE/Qdot655 MHC multimer combination, and persistence of an HLA-A1/CMV pp65$_{YSE}$ response, coded in the APC/Qdot605 MHC multimer combination. **a)** Staining directly *ex vivo*, no MAGE-A3$_{68\text{-}77}$-specific T cells detected and 0.10% HLA-A1/CMVpp65$_{YSE}$-specific T cells of 192.000 CD8 cells. **b)** Staining after an MHC multimer-based T-cell enrichment, 0.10% HLA-A1/MAGE-A3$_{68\text{-}77}$-specific T cells and 0.096% HLA-A1/CMV pp65$_{YSE}$-specific T cells of 229.000 CD8 cells. HLA-A1/CMV pp65$_{YSE}$ multimers were not included in the pool of MHC multimers used for the T-cell enrichment. The arrow indicates the enriched HLA-A1/MAGE-A3$_{68\text{-}77}$ response. Only the CD8 cells negative for all MHC multimers (gray) and specific for exactly two MHC multimers (black) are shown. PE, APC, Qdot605 and Qdot655 are streptavidin-conjugates.

Supplementary Figure 5

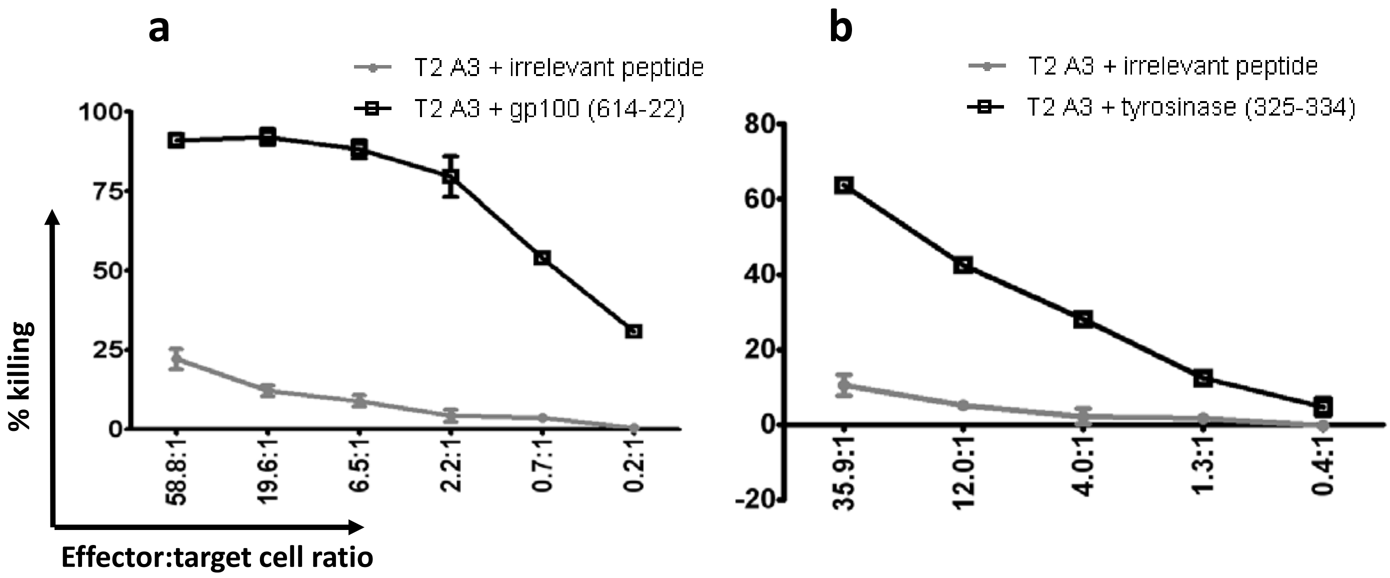

Supplementary Figure 5 a) Specific lysis of A3-T2 cells pulsed with $gp100_{614\text{-}22}$ upon co-culturing with a $gp100_{614\text{-}22}$-specific T-cell culture (60.0% of CD8) and no lysis of T2A3 cells pulsed with an irrelevant peptide. **b)** Specific lysis of A3-T2 cells pulsed with $tyrosinase_{325\text{-}334}$ upon co-culturing with a $tyrosinase_{325\text{-}334}$-specific T-cell culture (81.7% of CD8) and no lysis of T2A3 cells pulsed with an irrelevant peptide. Shown is the relative lysis of the target cells measured in a ^{51}Cr-release cytotoxicity assay; numbers indicate the MHC multimer + T cell:melanoma cell line ratio.

Supplementary Table 1. Overview of all peptides included in the study

Protein	Allele	Number	Position	C-terminalpos.	Sequence	Length	netMHCpan	NetMHC	Confirmed ligand	T-cell responses	VITAL-Far Red
gp100	HLA-A0101	1	143	151	WSQKRSFVY	9	0,573	0,519			
gp100	HLA-A0101	2	250	258	YLAEADLSY	9	0,478	0,542	1		
gp100	HLA-A0101	3	472	480	PLDCVLYRY	9	0,484	0,408	1		
gp100	HLA-A0301	4	17	25	ALLAVGATK	9	0,584	0,687	1		
gp100	HLA-A0301	5	34	42	GVSRQLRTK	9	0,443	0,485	1		
gp100	HLA-A0301	6	40	49	RTKAWNRQLY	10	0,433	0,409			
gp100	HLA-A0301	7	86	95	IALNFPGSQK	10	0,468	0,694	1		
gp100	HLA-A0301	8	87	95	ALNFPGSQK	9	0,648	0,785	1		
gp100	HLA-A0301	9	182	190	HTMEVTVYH	9	0,386	0,454	1		
gp100	HLA-A0301	10	182	191	HTMEVTVYHR	10	0,412	0,432			
gp100	HLA-A0301	11	187	195	TVYHRRGSR	9	0,367	0,634			
gp100	HLA-A0301	12	460	468	GTATLRLVK	9	0,588	0,648	1		
gp100	HLA-A0301	13	513	522	TVSCQGGLPK	10	0,435	0,666	1		
gp100	HLA-A0301	14	550	559	CQLVLHQILK	10	0,405	0,585			
gp100	HLA-A0301	15	551	559	QLVLHQILK	9	0,51	0,65	1		
gp100	HLA-A0301	16	609	617	VVLASLIYR	9	0,449	0,54	1		
gp100	HLA-A0301	17	609	618	VVLASLIYRR	10	0,371	0,372			
gp100	HLA-A0301	18	610	619	VLASLIYRRR	10	0,364	0,392			
gp100	HLA-A0301	19	613	622	SLIYRRRLMK	10	0,61	0,742			
gp100	HLA-A0301	20	614	622	LIYRRRLMK	9	0,735	0,811	1	3/14	Yes
gp100	HLA-A0301	21	634	642	SSHWLRLPR	9	0,398	0,435			
gp100	HLA-A1101	22	16	25	GALLAVGATK	10	0,546	0,579			
gp100	HLA-A1101	23	17	25	ALLAVGATK	9	0,605	0,716	1		
gp100	HLA-A1101	24	20	28	AVGATKVPR	9	0,529	0,563	1	1/5	Yes
gp100	HLA-A1101	25	34	42	GVSRQLRTK	9	0,57	0,563	1		
gp100	HLA-A1101	26	40	49	RTKAWNRQLY	10	0,461	0,43	1		
gp100	HLA-A1101	27	86	95	IALNFPGSQK	10	0,647	0,664	1		
gp100	HLA-A1101	28	87	95	ALNFPGSQK	9	0,634	0,731	1		
gp100	HLA-A1101	29	182	190	HTMEVTVYH	9	0,674	0,468	1		
gp100	HLA-A1101	30	182	191	HTMEVTVYHR	10	0,75	0,696			

gp100	HLA-A1101	31	183	192	TMEVTVYHRR	10	0,386	0,436			
gp100	HLA-A1101	32	186	195	VTVYHRRGSR	10	0,369	0,417	1		
gp100	HLA-A1101	33	187	195	TVYHRRGSR	9	0,414	0,708			
gp100	HLA-A1101	34	420	429	TTEWVETTAR	10	0,414	0,468			
gp100	HLA-A1101	35	460	468	GTATLRLVK	9	0,797	0,783	1		
gp100	HLA-A1101	36	460	469	GTATLRLVKR	10	0,589	0,559			
gp100	HLA-A1101	37	513	522	TVSCQGGLPK	10	0,678	0,783			
gp100	HLA-A1101	38	514	522	VSCQGGLPK	9	0,604	0,738	1		
gp100	HLA-A1101	39	550	559	CQLVLHQILK	10	0,576	0,57			
gp100	HLA-A1101	40	551	559	QLVLHQILK	9	0,529	0,579			
gp100	HLA-A1101	41	608	616	AVVLASLIY	9	0,388	0,483			
gp100	HLA-A1101	42	608	617	AVVLASLIYR	10	0,622	0,65			
gp100	HLA-A1101	43	609	617	VVLASLIYR	9	0,729	0,749	1		
gp100	HLA-A1101	44	609	618	VVLASLIYRR	10	0,62	0,731			
gp100	HLA-A1101	45	610	618	VLASLIYRR	9	0,449	0,536			
gp100	HLA-A1101	46	613	622	SLIYRRRLMK	10	0,385	0,74			
gp100	HLA-A1101	47	614	622	LIYRRRLMK	9	0,719	0,759	1		
gp100	HLA-A1101	48	633	642	SSSHWLRLPR	10	0,69	0,718			
gp100	HLA-A1101	49	634	642	SSHWLRLPR	9	0,709	0,741			
gp100	HLA-B0702	50	26	35	VPRNQDWLGV	10	0,47	0,427	1	1/18	ND
gp100	HLA-B0702	51	49	58	YPEWTEAQRL	10	0,402	0,499	1		
gp100	HLA-B0702	52	176	184	RAMLGTHTM	9	0,574	0,592	1		
gp100	HLA-B0702	53	198	206	VPLAHSSSA	9	0,696	0,567	1		
gp100	HLA-B0702	54	198	207	VPLAHSSSAF	10	0,789	0,745	1		
gp100	HLA-B0702	55	213	222	VPFSVSVSQL	10	0,575	0,569	1		
gp100	HLA-B0702	56	284	292	GPVTAQVVL	9	0,559	0,602	1		
gp100	HLA-B0702	57	314	323	RPTAEAPNTT	10	0,438	0,479			
gp100	HLA-B0702	58	319	327	APNTTAGQV	9	0,478	0,568	1	1/18	ND
gp100	HLA-B0702	59	362	371	APVQMPTAES	10	0,371	0,504			
gp100	HLA-B0702	60	366	374	MPTAESTGM	9	0,619	0,665			
gp100	HLA-B0702	61	366	375	MPTAESTGMT	10	0,391	0,52			
gp100	HLA-B0702	62	394	403	TPEATGMTPA	10	0,393	0,509	1		

gp100	HLA-B0702	63	401	409	TPAEVSIVV	9	0,445	0,554	1		
gp100	HLA-B0702	64	401	410	TPAEVSIVVL	10	0,558	0,678	1		
gp100	HLA-B0702	65	455	463	GPLLDGTAT	9	0,387	0,454			
gp100	HLA-B0702	66	455	464	GPLLDGTATL	10	0,59	0,641			
gp100	HLA-B0702	67	465	473	RLVKRQVPL	9	0,459	0,44	1		
gp100	HLA-B0702	68	502	510	VPSGEGDAF	9	0,416	0,628			
gp100	HLA-B0702	69	545	554	LPSPACQLVL	10	0,678	0,597	1		
gp100	HLA-B0702	70	596	604	VPLIVGILL	9	0,376	0,501			
gp100	HLA-B0702	71	627	635	VPQLPHSSS	9	0,41	0,427			
gp100	HLA-B0702	72	630	638	LPHSSSHWL	9	0,706	0,62	1		
gp100	HLA-B0702	73	640	649	LPRIFCSCPI	10	0,721	0,751			
gp100	HLA-B0702	74	647	655	CPIGENSPL	9	0,596	0,628	1		
gp100	HLA-B0702	75	647	656	CPIGENSPLL	10	0,417	0,388	1		
gp100	HLA-B0702	76	653	661	SPLLSGQQV	9	0,46	0,511	1		
MAGE-A3	HLA-A0101	77	68	77	ASSLPTTMNY	10	0,558	0,528	1	4/17	ND
MAGE-A3	HLA-A0101	78	69	77	SSLPTTMNY	9	0,379	0,384			
MAGE-A3	HLA-A0101	79	137	145	GSVVGNWQY	9	0,398	0,428			
MAGE-A3	HLA-A0101	80	168	176	EVDPIGHLY	9	0,742	0,651	1		
MAGE-A3	HLA-A0101	81	178	186	FATCLGLSY	9	0,414	0,398			
MAGE-A3	HLA-A0101	82	246	255	LTQHFVQENY	10	0,527	0,586			
MAGE-A3	HLA-A0101	83	250	258	FVQENYLEY	9	0,523	0,512	1	2/17	ND
MAGE-A3	HLA-A0301	84	115	123	ELVHFLLLK	9	0,427	0,558			
MAGE-A3	HLA-A0301	85	116	124	LVHFLLLKY	9	0,488	0,553			
MAGE-A3	HLA-A0301	86	144	153	QYFFPVIFSK	10	0,424	0,653			
MAGE-A3	HLA-A0301	87	145	153	YFFPVIFSK	9	0,484	0,584			
MAGE-A3	HLA-A0301	88	189	198	LLGDNQIMPK	10	0,565	0,669			
MAGE-A3	HLA-A0301	89	276	285	RALVETSYVK	10	0,474	0,589			
MAGE-A3	HLA-A0301	90	277	285	ALVETSYVK	9	0,496	0,382	1		
MAGE-A3	HLA-A0301	91	283	292	YVKVLHHMVK	10	0,374	0,565			
MAGE-A3	HLA-A0301	92	292	301	KISGGPHISY	10	0,475	0,583			
MAGE-A3	HLA-A1101	93	68	77	ASSLPTTMNY	10	0,492	0,588	1		
MAGE-A3	HLA-A1101	94	69	77	SSLPTTMNY	9	0,624	0,636	1		

MAGE-A3	HLA-A1101	95	115	123	ELVHFLLLK	9	0,523	0,553		
MAGE-A3	HLA-A1101	96	116	124	LVHFLLLKY	9	0,478	0,44		
MAGE-A3	HLA-A1101	97	116	125	LVHFLLLKYR	10	0,447	0,447		
MAGE-A3	HLA-A1101	98	144	153	QYFFPVIFSK	10	0,582	0,638		1
MAGE-A3	HLA-A1101	99	145	153	YFFPVIFSK	9	0,606	0,588		
MAGE-A3	HLA-A1101	100	189	198	LLGDNQIMPK	10	0,595	0,564		1
MAGE-A3	HLA-A1101	101	226	234	SVLEVFEGR	9	0,537	0,637		1
MAGE-A3	HLA-A1101	102	276	285	RALVETSYVK	10	0,612	0,674		1
MAGE-A3	HLA-A1101	103	277	285	ALVETSYVK	9	0,569	0,653		1
MAGE-A3	HLA-A1101	104	283	292	YVKVLHHMVK	10	0,478	0,561		
MAGE-A3	HLA-A1101	105	292	301	KISGGPHISY	10	0,454	0,464		1
MAGE-A3	HLA-B0702	106	64	73	SPQGASSLPT	10	0,556	0,596		1
MAGE-A3	HLA-B0702	107	71	79	LPTTMNYPL	9	0,737	0,697		1
MAGE-A3	HLA-B0702	108	125	133	RAREPVTKA	9	0,394	0,389		1
MAGE-A3	HLA-B0702	109	147	155	FPVIFSKAS	9	0,362	0,395		
MAGE-A3	HLA-B0702	110	147	156	FPVIFSKASS	10	0,369	0,453		
MAGE-A3	HLA-B0702	111	196	204	MPKAGLLII	9	0,566	0,531		1
MAGE-A3	HLA-B0702	112	196	205	MPKAGLLIIV	10	0,438	0,454		
MAGE-A3	HLA-B0702	113	216	225	APEEKIWEEL	10	0,398	0,481		
MAGE-A3	HLA-B0702	114	274	283	GPRALVETSY	10	0,375	0,365		
MAGE-A3	HLA-B0702	115	296	304	GPHISYPPL	9	0,626	0,586		1
MAGE-A3	HLA-B0702	116	301	309	YPPLHEWVL	9	0,417	0,443		
Mart1	HLA-A0101	117	107	116	LSAEQSPPPY	10	0,605	0,399		1
Mart1	HLA-A0301	118	7	15	HFIYGYPKK	9	0,372	0,411		
Mart1	HLA-A1101	119	5	14	DAHFIYGYPK	10	0,38	0,426		
Mart1	HLA-A1101	120	6	14	AHFIYGYPK	9	0,478	0,446		
Mart1	HLA-A1101	121	65	73	GTQCALTRR	9	0,492	0,542		1
Mart1	HLA-B0702	122	1	9	MPREDAHFI	9	0,642	0,61		1
Mart1	HLA-B0702	123	12	21	YPKKGHGHSY	10	0,493	0,486		
Mart1	HLA-B0702	124	49	57	RRRNGYRAL	9	0,395	0,593		
NY-ESO-1	HLA-A1101	125	127	136	TVSGNILTIR	10	0,528	0,565		
NY-ESO-1	HLA-B0702	126	44	53	GPRGAGAARA	10	0,615	0,571		1

NY-ESO-1	HLA-B0702	127	60	68	APRGPHGGA	9	0,589	0,621	1			
NY-ESO-1	HLA-B0702	128	60	69	APRGPHGGAA	10	0,771	0,772	1			
NY-ESO-1	HLA-B0702	129	63	72	GPHGGAASGL	10	0,61	0,502				
NY-ESO-1	HLA-B0702	130	82	90	GPESRLLEF	9	0,541	0,557	1	3/18	ND	
NY-ESO-1	HLA-B0702	131	113	122	APPLPVPGVL	10	0,484	0,557	1			
TRP-2	HLA-A0101	132	288	296	SLDDYNHLV	9	0,378	0,451				
TRP-2	HLA-A0101	133	398	406	FTDAIFDEW	9	0,488	0,391				
TRP-2	HLA-A0101	134	398	407	FTDAIFDEWM	10	0,619	0,416				
TRP-2	HLA-A0101	135	446	455	LTSDQLGYSY	10	0,739	0,655	1			
TRP-2	HLA-A0101	136	447	455	TSDQLGYSY	9	0,809	0,761	1			
TRP-2	HLA-A0301	137	8	16	FLLSCLGCK	9	0,452	0,64	1			
TRP-2	HLA-A0301	138	29	38	CMTVDSLVNK	10	0,475	0,522				
TRP-2	HLA-A0301	139	30	38	MTVDSLVNK	9	0,569	0,521	1			
TRP-2	HLA-A0301	140	141	149	FLGALDLAK	9	0,475	0,474	1			
TRP-2	HLA-A0301	141	141	150	FLGALDLAKK	10	0,411	0,443				
TRP-2	HLA-A0301	142	301	309	GTYEGLLRR	9	0,442	0,521	1			
TRP-2	HLA-A0301	143	306	314	LLRRNQMGR	9	0,403	0,425	1			
TRP-2	HLA-A0301	144	315	323	NSMKLPTLK	9	0,519	0,611				
TRP-2	HLA-A0301	145	401	409	AIFDEWMKR	9	0,418	0,48				
TRP-2	HLA-A0301	146	428	436	RMYNMVPFF	9	0,426	0,465				
TRP-2	HLA-A0301	147	488	496	VLLAFLQYR	9	0,419	0,546				
TRP-2	HLA-A0301	148	488	497	VLLAFLQYRR	10	0,387	0,473				
TRP-2	HLA-A0301	149	492	500	FLQYRRLRK	9	0,556	0,711				
TRP-2	HLA-A0301	150	504	513	PLMETHLSSK	10	0,472	0,582				
TRP-2	HLA-A0301	151	505	513	LMETHLSSK	9	0,556	0,628	1	1/14		ND
TRP-2	HLA-A1101	152	8	16	FLLSCLGCK	9	0,392	0,519	1			
TRP-2	HLA-A1101	153	29	38	CMTVDSLVNK	10	0,6	0,551	1			
TRP-2	HLA-A1101	154	30	38	MTVDSLVNK	9	0,871	0,809	1			
TRP-2	HLA-A1101	155	102	110	AGYNCGDCK	9	0,391	0,364	1			
TRP-2	HLA-A1101	156	141	149	FLGALDLAK	9	0,383	0,43				
TRP-2	HLA-A1101	157	180	189	SVYDFFVWLH	10	0,635	0,723				
TRP-2	HLA-A1101	158	196	205	TLLGPGRPYR	10	0,433	0,478				

TRP-2	HLA-A1101	159	301	309	GTYEGLLRR	9	0,653	0,704	1		
TRP-2	HLA-A1101	160	310	318	NQMGRNSMK	9	0,393	0,567	1		
TRP-2	HLA-A1101	161	314	323	RNSMKLPTLK	10	0,41	0,408			
TRP-2	HLA-A1101	162	315	323	NSMKLPTLK	9	0,777	0,739			
TRP-2	HLA-A1101	163	340	348	FQNSTFSFR	9	0,426	0,498			
TRP-2	HLA-A1101	164	401	409	AIFDEWMKR	9	0,659	0,721	1		
TRP-2	HLA-A1101	165	446	455	LTSDQLGYSY	10	0,437	0,436	1		
TRP-2	HLA-A1101	166	487	496	FVLLAFLQYR	10	0,437	0,511			
TRP-2	HLA-A1101	167	488	496	VLLAFLQYR	9	0,511	0,577			
TRP-2	HLA-A1101	168	488	497	VLLAFLQYRR	10	0,459	0,572			
TRP-2	HLA-A1101	169	489	497	LLAFLQYRR	9	0,39	0,417			
TRP-2	HLA-A1101	170	492	500	FLQYRRLRK	9	0,388	0,572			
TRP-2	HLA-A1101	171	504	513	PLMETHLSSK	10	0,449	0,523	1		
TRP-2	HLA-A1101	172	505	513	LMETHLSSK	9	0,51	0,527	1		
TRP-2	HLA-B0702	173	2	10	SPLWWGFLL	9	0,497	0,602	1	2/18	No
TRP-2	HLA-B0702	174	25	34	FPRVCMTVDS	10	0,427	0,449			
TRP-2	HLA-B0702	175	41	49	CPRLGAESA	9	0,593	0,594	1		
TRP-2	HLA-B0702	176	69	77	RPWSGPYIL	9	0,735	0,72	1		
TRP-2	HLA-B0702	177	86	94	WPRKFFHRT	9	0,581	0,504			
TRP-2	HLA-B0702	178	86	95	WPRKFFHRTC	10	0,572	0,589			
TRP-2	HLA-B0702	179	125	133	VIRQNIHSL	9	0,585	0,59	1		
TRP-2	HLA-B0702	180	134	142	SPQEREQFL	9	0,492	0,555	1		
TRP-2	HLA-B0702	181	173	181	QPQFANCSV	9	0,413	0,555	1	1/18	ND
TRP-2	HLA-B0702	182	199	207	GPGRPYRAI	9	0,498	0,57			
TRP-2	HLA-B0702	183	382	391	LPHSAANDPI	10	0,625	0,529	1		
TRP-2	HLA-B0702	184	466	474	TPGWPTTLL	9	0,496	0,68	1	2/18	ND
TRP-2	HLA-B0702	185	469	477	WPTTLLVVM	9	0,606	0,614	1	1/18	ND
TRP-2	HLA-B0702	186	497	505	RLRKGYTPL	9	0,725	0,633	1		
TRP-2	HLA-B0702	187	497	506	RLRKGYTPLM	10	0,578	0,39	1	1/18	No
Tyrosinase	HLA-A0101	188	247	256	CTDEYMGGQH	10	0,518	0,49			
Tyrosinase	HLA-A0101	189	360	369	SSMHNALHIY	10	0,588	0,53			
Tyrosinase	HLA-A0101	190	439	447	FISSKDLGY	9	0,532	0,441			

Tyrosinase	HLA-A0101	191	440	449	ISSKDLGYDY	10	0,591	0,446			
Tyrosinase	HLA-A0101	192	454	463	DSDPDSFQDY	10	0,597	0,664	1		
Tyrosinase	HLA-A0301	193	24	33	CVSSKNLMEK	10	0,531	0,645	1		
Tyrosinase	HLA-A0301	194	25	33	VSSKNLMEK	9	0,535	0,56	1		
Tyrosinase	HLA-A0301	195	95	104	FMGFNCGNCK	10	0,401	0,477	1		
Tyrosinase	HLA-A0301	196	122	131	NIFDLSAPEK	10	0,51	0,5	1		
Tyrosinase	HLA-A0301	197	133	142	KFFAYLTLAK	10	0,681	0,636			
Tyrosinase	HLA-A0301	198	134	142	FFAYLTLAK	9	0,449	0,512	1		
Tyrosinase	HLA-A0301	199	215	224	LLRWEQEIQK	10	0,497	0,451	1	1/14	ND
Tyrosinase	HLA-A0301	200	325	334	TQYESGSMDK	10	0,567	0,567	1	1/14	Yes
Tyrosinase	HLA-A0301	201	361	369	SMHNALHIY	9	0,428	0,402			
Tyrosinase	HLA-A0301	202	395	403	SIFEQWLRR	9	0,506	0,564	1		
Tyrosinase	HLA-A0301	203	426	434	MVPFIPLYR	9	0,384	0,516			
Tyrosinase	HLA-A0301	204	465	473	KSYLEQASR	9	0,416	0,51	1		
Tyrosinase	HLA-A0301	205	494	503	GLVSLLCRHK	10	0,464	0,597			
Tyrosinase	HLA-A0301	206	495	503	LVSLLCRHK	9	0,416	0,385			
Tyrosinase	HLA-A0301	207	496	505	VSLLCRHKRK	10	0,374	0,49			
Tyrosinase	HLA-A0301	208	497	505	SLLCRHKRK	9	0,52	0,606	1		
Tyrosinase	HLA-A1101	209	14	22	QTSAGHFPR	9	0,663	0,704	1		
Tyrosinase	HLA-A1101	210	24	33	CVSSKNLMEK	10	0,748	0,72	1		
Tyrosinase	HLA-A1101	211	25	33	VSSKNLMEK	9	0,752	0,751	1		
Tyrosinase	HLA-A1101	212	95	104	FMGFNCGNCK	10	0,375	0,373	1		
Tyrosinase	HLA-A1101	213	96	104	MGFNCGNCK	9	0,569	0,363	1		
Tyrosinase	HLA-A1101	214	122	131	NIFDLSAPEK	10	0,656	0,723	1		
Tyrosinase	HLA-A1101	215	133	142	KFFAYLTLAK	10	0,685	0,692			
Tyrosinase	HLA-A1101	216	134	142	FFAYLTLAK	9	0,459	0,379			
Tyrosinase	HLA-A1101	217	231	239	FTIPYWDWR	9	0,395	0,401	1		
Tyrosinase	HLA-A1101	218	270	278	SSWQIVCSR	9	0,571	0,458			
Tyrosinase	HLA-A1101	219	274	282	IVCSRLEEY	9	0,364	0,446			
Tyrosinase	HLA-A1101	220	325	334	TQYESGSMDK	10	0,672	0,746			
Tyrosinase	HLA-A1101	221	360	369	SSMHNALHIY	10	0,553	0,643			
Tyrosinase	HLA-A1101	222	361	369	SMHNALHIY	9	0,453	0,39			

Tyrosinase	HLA-A1101	223	380	389	SANDPIFLLH	10	0,448	0,538			
Tyrosinase	HLA-A1101	224	395	403	SIFEQWLRR	9	0,696	0,742			
Tyrosinase	HLA-A1101	225	425	434	YMVPFIPLYR	10	0,478	0,454	1		
Tyrosinase	HLA-A1101	226	426	434	MVPFIPLYR	9	0,648	0,633	1		
Tyrosinase	HLA-A1101	227	434	443	RNGDFFISSK	10	0,468	0,395			
Tyrosinase	HLA-A1101	228	465	473	KSYLEQASR	9	0,483	0,537			
Tyrosinase	HLA-A1101	229	494	503	GLVSLLCRHK	10	0,4	0,475			
Tyrosinase	HLA-A1101	230	495	503	LVSLLCRHK	9	0,492	0,55			
Tyrosinase	HLA-A1101	231	496	505	VSLLCRHKRK	10	0,525	0,619			
Tyrosinase	HLA-A1101	232	497	505	SLLCRHKRK	9	0,497	0,594			
Tyrosinase	HLA-B0702	233	20	28	FPRACVSSK	9	0,522	0,441	1		
Tyrosinase	HLA-B0702	234	63	71	APLGPQFPF	9	0,572	0,56	1		
Tyrosinase	HLA-B0702	235	109	118	GPNCTERRLL	10	0,537	0,489	1		
Tyrosinase	HLA-B0702	236	151	159	IPIGTYGQM	9	0,529	0,583	1		
Tyrosinase	HLA-B0702	237	204	213	APAFLPWHRL	10	0,57	0,629	1		
Tyrosinase	HLA-B0702	238	208	216	LPWHRLFLL	9	0,585	0,727			
Tyrosinase	HLA-B0702	239	259	268	NPNLLSPASF	10	0,526	0,608			
Tyrosinase	HLA-B0702	240	264	272	SPASFFSSW	9	0,492	0,648	1	1/18	ND
Tyrosinase	HLA-B0702	241	309	317	TPRLPSSAD	9	0,41	0,561	1		
Tyrosinase	HLA-B0702	242	309	318	TPRLPSSADV	10	0,643	0,674	1	1/18	ND
Tyrosinase	HLA-B0702	243	312	320	LPSSADVEF	9	0,508	0,429			
Tyrosinase	HLA-B0702	244	335	344	AANFSFRNTL	10	0,365	0,482			
Tyrosinase	HLA-B0702	245	430	438	IPLYRNGDF	9	0,536	0,609	1		
Tyrosinase	HLA-B0702	246	430	439	IPLYRNGDFF	10	0,48	0,575			
Tyrosinase	HLA-B0702	247	480	488	GAAMVGAVL	9	0,411	0,451	1		
Tyrosinase	HLA-B0702	248	507	515	LPEEKQPLL	9	0,383	0,368	1		
Tyrosinase	HLA-B0702	249	507	516	LPEEKQPLLM	10	0,446	0,444	1		

NetMHC and netMHCpan predict the MHC-peptide affinity. Prediction value = 1 - $\log_{50.000}$(IC50). Prediction value 0.3617 equals an IC50 of 1000 nM. Higher prediction value means higher affinity (lower number). Ligands were confirmed by use of MHC ELISA (Ref. 25) (1 = confirmed ligand) and T-cell responses detected upon staining with combinatorial encoded MHC multimers (Ref. 27). Selected cultures were tested in VITAL-Far Red and Cr51 release cytotoxicity assays with electroporated, HLA-transduced K562 cells and cells from a melanoma cell line, respectively.
